# Marine Scrubbers vs Low-Sulfur Fuels: A Comprehensive Well-To-Wake Life Cycle Assessment Supported by Measurements Aboard an Ocean-Going Vessel

**DOI:** 10.1021/acs.est.4c10006

**Published:** 2025-04-04

**Authors:** Patritsia M. Stathatou, Ievgenii Petrunia, Torsten Barenthin, George Gotsis, Paul Jeffrey, Christopher Fee, Scott Bergeron, Marios Tsezos, Michael Triantafyllou, Neil Gershenfeld

**Affiliations:** † School of Chemical and Biomolecular Engineering, Georgia Institute of Technology, Atlanta, Georgia 30332, United States; ‡ Renewable Bioproducts Institute, Georgia Institute of Technology, Atlanta, Georgia 30332, United States; § Center for Bits and Atoms, Massachusetts Institute of Technology, Cambridge, Massachusetts 02139, United States; ∥ Oldendorff Carriers GmbH & Co. KG., Willy-Brandt-Allee 6, Lübeck 23554, Germany; ⊥ Naias Laboratories, S.A., Piraeus 185 40, Greece; # Laboratory of Environmental Science and Engineering, School of Mining and Metallurgical Engineering, National Technical University of Athens, Athens 15773, Greece; ∇ Department of Mechanical Engineering, Massachusetts Institute of Technology, Cambridge, Massachusetts 02139, United States

**Keywords:** marine scrubbers, exhaust gas cleaning systems, well-to-tank impacts, well-to-wake impacts, washwater discharge, LCA

## Abstract

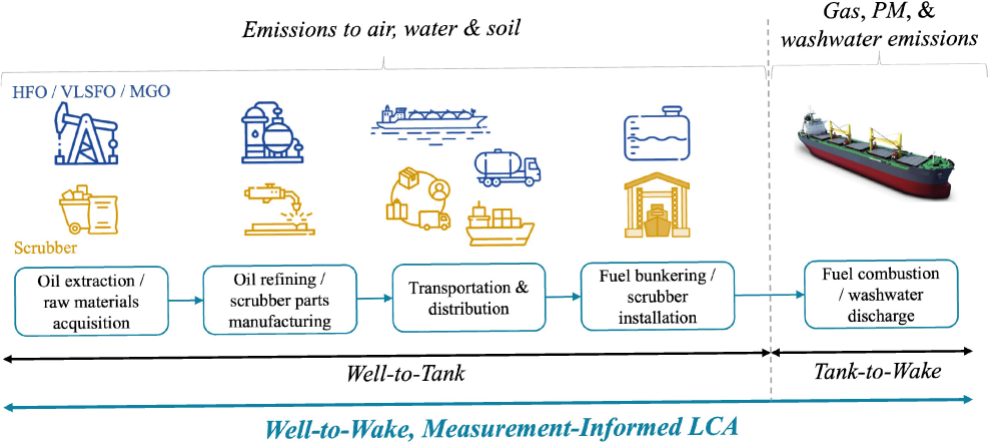

Maritime transport significantly contributes to global emissions, prompting the International Maritime Organization to implement stricter regulations to reduce pollution. Since 2020, fuel sulfur (S) content limits have been reduced, requiring either the use of low-S fuels or the installation of marine scrubbers to continue using heavy fuel oil (HFO). While scrubbers are a widely adopted solution for reducing S emissions, their benefits are controversial and uncertainty remains regarding scrubber environmental impacts and their appropriate evaluation. Here, we systematically assess the environmental impacts of scrubbers operating on HFO to those of low-S fuels across various categories, through a measurement-informed Well-to-Wake (WtW) life cycle assessment (LCA). Gaseous and particulate matter (PM) emissions data were collected while a bulk carrier vessel was burning 3% S HFO, 0.1% S marine gas oil (MGO), and 0.5% S very low-S fuel oil (VLSFO) under similar engine operating modes during an actual ocean voyage. Seawater and washwater samples were also analyzed, alongside fuel, cylinder oil, and lubricant samples. The results suggest that, in various instances the use of HFO with a scrubber can be considered equivalent to MGO use while outperforming VLSFO use from a WtW perspective, for large, ocean-going bulk carrier vessels in open seas. These findings indicate that end-of-pipe solutions may not always be inferior to start-of-pipe alternatives, underscoring the need for comprehensive LCA studies to properly assess emission abatement technologies.

## Abstract 2

There is uncertainty regarding the environmental impacts of marine scrubbers. This study demonstrates that scrubbers operating on heavy fuel oil can match or surpass low-sulfur fuel performance for large, ocean-going bulk carrier vessels in open seas, through a comprehensive, measurement-informed LCA assessment.

## 1 Introduction

Maritime transport is vital to the global economy, handling over 80% of international trade by volume.^1^ At the same time, it contributes around 3% of global annual greenhouse gas (GHG) emissions, alongside 4–9% of sulfur dioxide (SO_2_) and 15% of nitrogen oxides (NOx) emissions.^2^ Moreover, shipping generates fine particulate matter (PM_2.5_), which negatively impacts air quality and human health.^3^ Efforts to reduce maritime pollution have led to stricter regulations. The International Maritime Organization (IMO), among other measures, established Emission Control Areas (ECAs), where NOx and SOx emissions are rigorously controlled, and set sulfur (S) content limits for marine fuels, reducing the maximum allowable S content to 0.1% m/m in ECAs and 0.5% m/m outside ECAs, since January 1, 2020.^4,5^

Bulk carrier vessels, representing 22% of the global merchant fleet,^6^ are key contributors to shipping emissions.^7,8^ They typically burn heavy fuel oil (HFO), a residual fossil fuel with higher S (1–3%), ash, metals, and water contents than distillate fuels, such as marine gas oil (MGO), leading to higher SO_*x*_ and PM emissions.^2,9^ To meet the recent S fuel limits, bulk carriers can switch to low-S fossil fuels, such as MGO and very low-S fuel oil (VLSFO), or burn alternative fuels, like biofuels, which have low S contents.^8^ An alternative option would be to implement exhaust gas cleaning systems or scrubbers to remove SO_*x*_ emissions, while continuing burning HFO. Low-S fossil and alternative fuels are considerably more expensive, while the current availability of alternative fuels is low to accommodate demand. Therefore, the third option is the most widely adopted, with over 5,800 vessels equipped with scrubbers in 2024^10^ compared to less than 800 in 2018.^2,11^

Scrubbers, commonly used in land-based facilities like power plants, remove SO_x_ from exhaust gases either through liquid absorption (wet scrubbers) or solid binding (dry scrubbers). While dry scrubbers are rarely used on ships due to weight and space issues, wet marine scrubbers are more prevalent and include open-loop, closed-loop, and hybrid systems.^12^ Open-loop systems, which use seawater and discharge washwater into the sea, are the most common due to their simplicity and lower cost.^2,9^

While open-loop marine scrubbers effectively reduce SO_*x*_ emissions, concerns about the discharge of washwater into the sea are growing. The acidic nature of washwater, coupled with the presence of organic and inorganic substances, like heavy metals and polycyclic aromatic hydrocarbons (PAHs), raise questions about potential impacts on marine life.^11,13^ Although IMO regulates scrubber washwater discharge setting limits for pH, turbidity, nitrates, and PAHs, it does not address other pollutants like heavy metals.^13,14^ IMO is working on revising current limits,^15^ but such efforts need to be supported by scientific evidence.

Several studies have monitored marine fuels emissions,^16−19^ while efforts to assess the impacts of scrubber washwater on marine life have been reported.^20−24^ In addition, life cycle assessments (LCA) of fuels and scrubbers have been conducted.^12,25,26^ Despite various analyses, uncertainty persists regarding the environmental effects of marine scrubbers, particularly in determining the most relevant impacts, their magnitude, and appropriate evaluation methods.^11,17,27,28^ Existing LCA studies lack onboard measurements or focus on specific impact categories, such as climate change, and specific subcomponents of the analyzed systems without accounting for all the stages involved. Holistic, measurement-informed LCAs accounting for the potential environmental impacts associated with HFO and scrubber production and operation, versus those of low-S fuels are missing.

**Figure 1:**
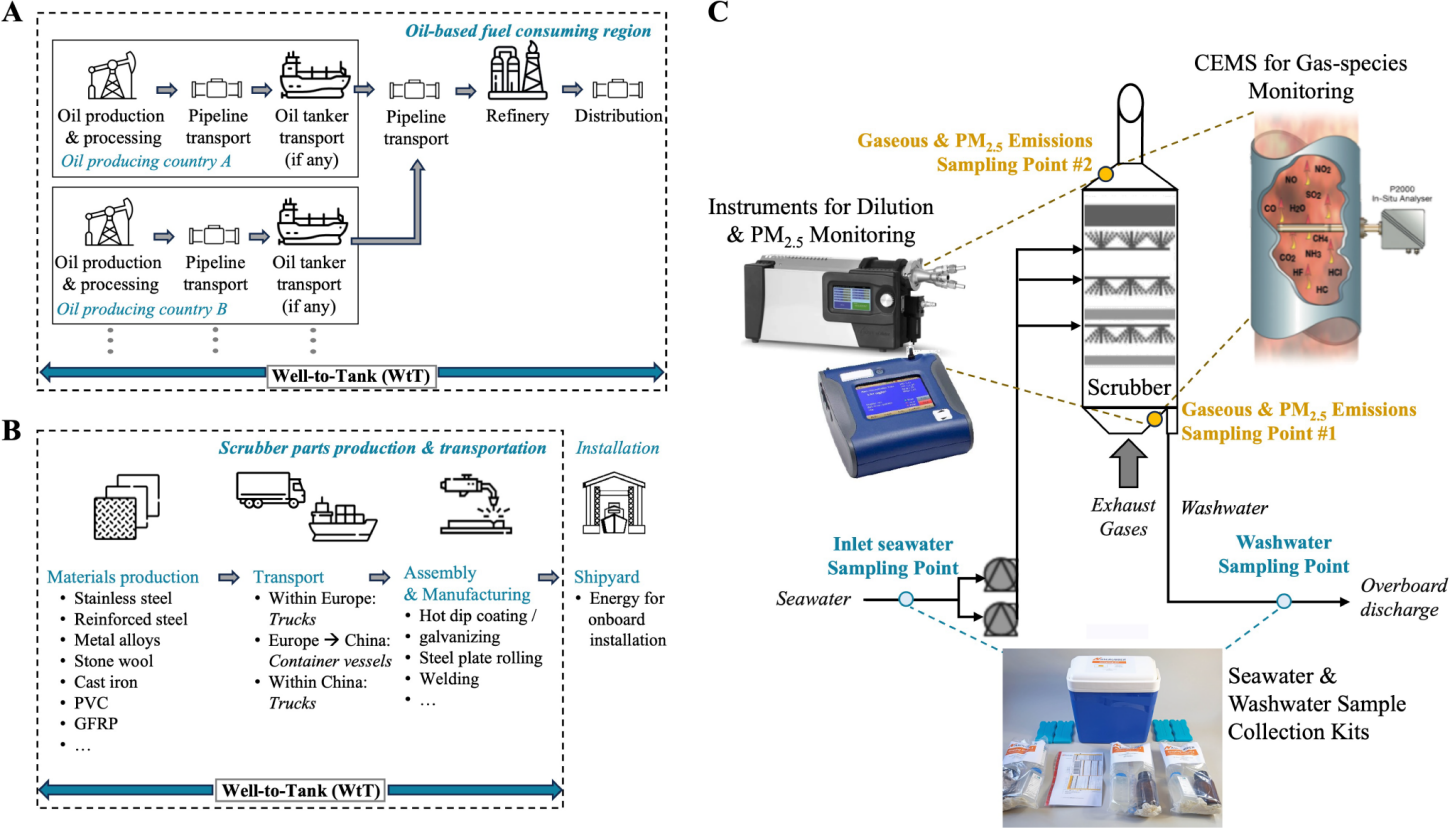
WtT considerations and onboard measurements. (A) The WtT system boundaries of the considered fuels, including all stages from oil extraction up to fuel bunkering aboard the vessel. (B) The WtT system boundaries of the scrubber, including all stages from raw materials production up to the installation of the scrubber aboard the vessel at the shipyard; the shipyard stage is not included in the analysis. (C) Schematic diagram of the onboard emission monitoring systems. (Images adapted or sourced from refs.^38−43^)

To provide a robust assessment of the environmental impacts of using scrubbers with HFO versus low-S fuels, we conducted a comprehensive Well-to-Wake (WtW) LCA, accounting for impacts from their sourcing, production, conversion, transport, distribution, and eventual use onboard the vessel.^29^ This analysis combines a Well-to-Tank (WtT) component, which concerns all impacts from raw materials extraction up to the bunkering of the fuels aboard the vessel and/or the installation of the scrubber, with a Tank-to-Wake (TtW) component, resulting from the fuel combustion onboard the vessel and the corresponding use of the scrubber^29^ (Figure 1). We monitored air and washwater emissions aboard an ocean-going bulk carrier and compared HFO with scrubber, MGO, and VLSFO, across various impact categories. Gaseous and PM_2.5_ emissions were measured for all three fuels under similar engine operating modes, following the International Organization for Standardization (ISO) 8178 guidelines.^30,31^ In parallel, during scrubber operation, incoming seawater and outgoing washwater samples were analyzed for over 60 quality parameters.

## 2 Materials and Methods

### 2.1 LCA Methodology

The LCA followed ISO 14040^32^ and 14044.^33^ The functional unit of the analysis is one MJ of energy input to the vessel engines (MJ_in_) (detailed in SI Note 4).

Data from the ecoinvent v3.10 database^34^ were used for calculating the WtT impacts of the fuels and the scrubber. To assess the WtT impacts of the fuels, the ecoinvent data, originally provided per kg of fuel, were converted to the functional unit using the calorific value of each fuel (detailed in SI Note 1). For the scrubber, two WtT assessments were conducted: (a) per scrubber produced, and (b) per MJ_in_ over the scrubber’s 20-year lifetime. The second approach allowed the scrubber’s WtT impacts to be expressed in the same functional unit as that of the fuels, enabling direct comparison and integration of the calculated WtT impacts for the fuels and the scrubber.

The system boundaries for fuels WtT impacts covered all stages from oil extraction to fuel bunkering aboard the vessel (Figure 1A). Since ocean-going vessels bunker fuels from diverse global sources, average global impacts were considered,^35^ accounting for uncertainty due to variations in calorific values^36,37^ in the error margins (detailed description in SI Note 1).

The system boundaries for scrubber production covered all stages from raw material acquisition up to the installation of the scrubber aboard the vessel (Figure 1B). Data on all these stages were provided by the scrubber manufacturer. The main production materials used included steel and aluminum alloys, and were mostly sourced within Europe. Detailed information on the calculation of scrubber WtT impacts is provided in the Supporting Information (SI Note 2, Table S1, Figure S1).

TtW impacts were calculated based on data from onboard measurements (Figure 1C). The gaseous and PM_2.5_ emissions presented in Section 3.2 are reported in grams per kilowatt-hour of engine output (g/kW h) to align with standard reporting practices, while the washwater emissions are presented as concentrations to facilitate comparisons with existing literature and regulatory guidelines. To calculate TtW LCA impacts, these emissions were converted to the study’s functional unit, MJ_in_, considering an average engine efficiency as detailed in SI Note 3. By combining the WtT and TtW impacts, the WtW impacts were determined.

Impacts from climate change, photochemical ozone formation, terrestrial acidification, freshwater and marine eutrophication, freshwater, marine, and terrestrial ecotoxicity, and fine particulate matter formation were assessed, as expressed by the relevant midpoint impact indicators of the ReCiPe (2016) method.^44^ The IPCC (2021) characterization model^45^ was used for calculating climate change, while the ReCiPe (2016), v1.03, midpoint, no long-term, Hierarchist perspective was employed for calculating all other LCA indicators.

### 2.2 Onboard Emission Measurement Campaign

An onboard emissions measurement and sampling plan was developed following established protocols.^14,30,31,46−48^

#### 2.2.1 Vessel, Engines, and Scrubber System Description

The Hedwig Oldendorff, a representative ocean-going bulk carrier vessel, was selected for this study (further details in SI Note 5). It is equipped with a slow-speed, two-stroke diesel main engine (ME) of 15,131 kW nominal maximum continuous rating (MCR), which drives the propeller shaft directly, and three medium-speed, four-stroke auxiliary engines (AEs) of 980 kW rated output. Both the ME and the AEs are compliant with the IMO Tier II regulations for NOx emissions.^49^ The vessel’s engines are connected to an open-loop scrubber system (SI Note 5), using seawater to remove SO_2_ from the exhaust gases. The scrubber is designed to handle a maximum capacity of 85% of the ME’s MCR and 85% of the combined output of two AEs running in parallel. The SO_2_ removal efficiency depends on the flow rates of the exhaust gases and seawater, with no alkali addition.

#### 2.2.2 Tested Fuels and Voyage

The onboard sampling and measurements were conducted during a 6-day voyage. Fuels were bunkered in China. The voyage began at Taicang port and ended at Hong Kong. The vessel burned MGO while in Taicang and during departure, switched to VLSFO, and then HFO. Upon approaching Hong Kong, it switched back to MGO. Weather conditions remained consistent throughout the voyage.

Fuel samples were collected before the high-pressure pump and analyzed for chemical composition and physical properties. Samples of the ME cylinder oils (the same cylinder oil was used for VLSFO and HFO, and a different one for MGO) and the AE lubricant (same for all fuels) were also collected to close the mass balance. Detailed testing results are provided in the Supporting Information (SI Note 6, Tables S2–S4, Figures S4–S7).

The global average S content of HFO and MGO is 2.6% m/m and 0.07% m/m, respectively.^50^ The fuels tested in this study had S contents equal to or higher than the global averages to represent worst-case emission scenarios (HFO: 3% m/m; MGO: 0.1% m/m; VLSFO: 0.5% m/m). Additionally, the measured calorific values of HFO (40.41 MJ kg^–1^) and MGO (42.76 MJ kg^–1^) align with global averages.^35^ For VLSFO the global average calorific value was considered (41.62 MJ kg^–1^).^36,37^ The considered average VLSFO calorific value is also in excellent agreement with data from approximately 400 samples of VLSFO fuels used by Oldendorff’s vessels throughout 2024.

#### 2.2.3 Engine Operating Modes

Four engine operating modes were specified: idle, and at 25%, 50%, and 80% of the ME’s load (% MCR), in an effort to align with the steady-state discrete-mode test cycle E2 recommended by ISO 8178-4.^31^ However, operating at 100% load, as suggested by ISO 8178-4, was not feasible, since this load does not typically occur. Although the scrubber was designed for a maximum load of 85% MCR of the ME, with all three AEs running at 85% MCR in parallel, in real-life conditions the ME rarely exceeds 80% load, while only two of the three AEs run in parallel. In all four modes, the two AEs operated at 30–60% load, while the third one remained in standby mode.

Emission measurements were conducted for each fuel across the four engine modes in ascending order. Weighting factors for each mode were determined based on hourly engine output data (% MCR) for Hedwig Oldendorff (SI Note 7). The engine operating modes, their respective weighting factors, and recorded engine and scrubber conditions for the different fuels are presented in Table S5 and SI Note 7.

#### 2.2.4 Gaseous Monitoring

Concentrations of CO_2_, CO, NOx, and SO_2_ were measured on a wet basis using two P2000 Continuous Emission Monitoring Systems (CEMS)^39^ (Protea Ltd., United Kingdom) (detailed in SI Note 8). A Wöhler A 550 Industrial^51^ portable, nondispersive, infrared flue gas analyzer (Wöhler USA, Inc., Middleton, MA, USA) was used to verify the CEMS measurements. All instruments were calibrated per manufacturer specifications.

To ensure robust and valid measurements, we utilized two sampling points: one located upstream (before) and one downstream (after) the scrubber. For the tests with MGO and VLSFO, the scrubber was not in operation. In these cases, we took measurements from both sampling points and calculated the average to improve data reliability. For the tests with HFO, the scrubber was operational. Measurements were again taken from the same two sampling points to enable a direct comparison of emissions upstream and downstream of the scrubber, allowing us to evaluate the scrubber’s effectiveness (Figure 1C). Gas measurements complied with ISO 8178.^30,31^ The ME was brought to steady-state before measurements, which were recorded over 10–30 min.

#### 2.2.5 PM Monitoring

PM_2.5_ emissions were measured upstream of the scrubber for all the tested fuels, as well as downstream of the scrubber for HFO (Figure 1C), under the same engine conditions as the gas emissions (detailed in SI Note 8). PM_2.5_ mass readings were recorded for 10–30 min per mode, with the average values used.

The DustTrak DRX 8533EP aerosol monitor (TSI Inc., Minnesota, USA),^40^ which employs light-scattering laser photometry for real-time readings, was used for PM_2.5_ mass measurements. The DustTrak photometer, does not measure particles smaller than 100 nm in diameter and may underrepresent ultrafine particle contributions. Despite this limitation, it was chosen as the most practical tool that can provide valuable real-time data for comparing PM_2.5_ mass emissions across fuels under real-world conditions, given the challenges of performing gravimetric measurements onboard.

The exhaust gas was first passed through a Dekati eDiluter Pro system (Dekati, Kangasala, Finland),^41^ which applied two-stage dilution and conditioning. The first stage maintained a temperature of 260 °C for upstream and 200 °C for downstream of the scrubber to prevent particle loss and condensation^52−54^ (exhaust temperatures: ∼220 °C upstream; ∼30 °C downstream of the scrubber). At the second stage, ambient temperature air (<50 °C) was used to further dilute the sample and prevent condensation. The exhaust gas was diluted at a 5:1 ratio in both stages.

A Dekati DI-1010b pressurized air drying and filtration unit conditioned the vessel’s pressurized air used for dilution. Exhaust gas was collected using a perforated probe and transported via a heated sampling line to the eDiluter Pro, while a heated Dekati Cyclone removed coarse PM (>10 μm).

The PM_2.5_ measurement method used differs from the ISO 8178^30,31^ filter weighing method, although exhaust gas extraction and dilution comply with ISO guidelines. This real-time monitoring method was chosen for its efficiency and practicality, recognizing that ISO 8178^30^ allows alternative methods if equivalency is demonstrated. While we were unable to directly demonstrate equivalence with gravimetric methods, a prior study^18^ showed good agreement between the DustTrak photometer and the DMS500 for PM_1_ mass measurements, supporting the suitability of the DustTrak for real-time particulate monitoring. Given these factors and inherent limitations of the TSI DustTrak discussed above, the PM_2.5_ data should be interpreted as indicative rather than absolute. A correction factor has been applied to the measured PM_2.5_ data, further described in SI Note 11 together with calibration details.

#### 2.2.6 Seawater and Washwater Sample Collection and Preservation

Incoming seawater and washwater discharge samples were collected from onboard monitoring stations (Figures 1C, S11 and S12). Sampling occurred during the four engine operating modes described above, with ME and scrubber running in steady state. Within 15 min of sample collection, pH, turbidity, and total residual oxidants were measured. Samples were then preserved and stored appropriately until they were dispatched for chemical analyses. Additional information on sampling, preservation and storage procedures is provided in SI Note 9.

#### 2.2.7 Seawater and Washwater Analyses

Seawater and washwater samples were analyzed for over 60 chemical parameters, including all targeted substances recommended by the IMO.^55^ Specifically, the samples were tested for total dissolved and suspended solids (TDS and TSS respectively), nitrates (NO_3_^–^), nitrites (NO_2_^–^), ammonium (NH_4_^+^), total nitrogen (N), phosphates (PO_4_^3–^), total phosphorus (P), sulfates (SO_4_^2–^), sulfites (SO^_3_2–^), oil in water, the US EPA 16 priority PAHs,^56^ 1,4-dichlorobenzene, benzene, toluene, ethylbenzene, and xylene compounds (BTEX), total hydrocarbons C10–C40, and 23 metals. All the chemical parameters analyzed are shown in Table S6, while detailed analysis methods and procedures are provided in SI Note 10.

### 2.3 Calculation of Emission Factors (EFs)

Emission factors (EFs) were calculated following ISO 8178.^30,31^ Emissions were measured instantaneously at minute intervals. Instantaneous emissions were converted to grams per kilowatt hour (g kW^–1^ h^–1^) and normalized to standard conditions, using the carbon balance method.^31,57−59^ EFs for each engine mode were multiplied with the relevant weighting factors and summed together to provide the weighted average EFs. Additional information on EF calculation is provided in SI Note 11.

## 3 Results and Discussion

### 3.1 Well-To-Tank (WtT) Assessment

Scrubber production impacts are presented in Figure S15 and SI Note 12. The same trend is observed across all impact categories. The production of scrubber materials is the dominant contributor to the WtT impacts, in consistency with prior studies.^26^ Materials transportation ranks second in most categories, except for climate change, freshwater and marine eutrophication. Energy consumption for manufacturing was the least contributing factor to WtT impacts, with the exception of the three aforementioned impact categories.

Scrubber WtT impacts throughout its lifetime, i.e., per MJ of incoming energy to the vessel’s engines across 20 years of operation (∼ 6.3 billion MJ_in_), were found to be negligible compared to the corresponding impacts of fuel use, i.e., 0.035 g CO_2_-eq/MJ_in_ ± 0.002 compared to 16.8 g CO_2_-eq/MJ_in_ ± 1.5 for HFO (Figure S16).

Although the absolute mean values of the combined WtT impacts of scrubber and HFO are lower than those of MGO and VLSFO across all impact categories, this difference is statistically significant in only five of them: climate change, human health damage due to photochemical ozone formation, terrestrial acidification, terrestrial ecotoxicity, and fine PM formation (Figure S16). The combined WtT terrestrial acidification impacts of HFO and scrubber are 38% lower, while the photochemical ozone formation impacts are 15% lower compared to MGO and VLSFO. The reductions of HFO and scrubber in the other impact categories, fall within intermediate ranges, with WtT climate change impacts being 24% lower and fine PM formation potential being 34% lower.

Transportation and distribution accounts for less than 10% of the WtT impacts across all fuels and impact categories, with the majority of WtT impacts arising from the fuels’ production processes. The reduced WtT impacts of HFO are due to the less energy-intensive refining processes involved, generating fewer emissions, requiring lower resource consumption and generating less waste compared to low-S fuels.^35,60^ HFO is derived from the heavier fractions of crude oil, whereas MGO is produced from lighter fractions separated after fractional distillation, i.e., diesel and kerosene, and undergoes desulfurization and further treatment leading to higher impacts.^61,62^ VLSFO is usually a blend of HFO and MGO with varying ratios.^35,36^ However, VLSFO’s actual composition is more complex than a simple binary mixture of HFO and MGO, often containing various additives. These components can lead to variations in the fuel’s energy content. Although the impact of such additives has not been directly accounted for in these calculations, this uncertainty has been indirectly factored in by considering the range of VLSFO energy contents within the error margins of the WtT values.

### 3.2 Tank-To-Wake (TtW) Assessment

#### 3.2.1 Gaseous Emissions

##### 3.2.1.1 CO_2_ Emissions

**Figure 2:**
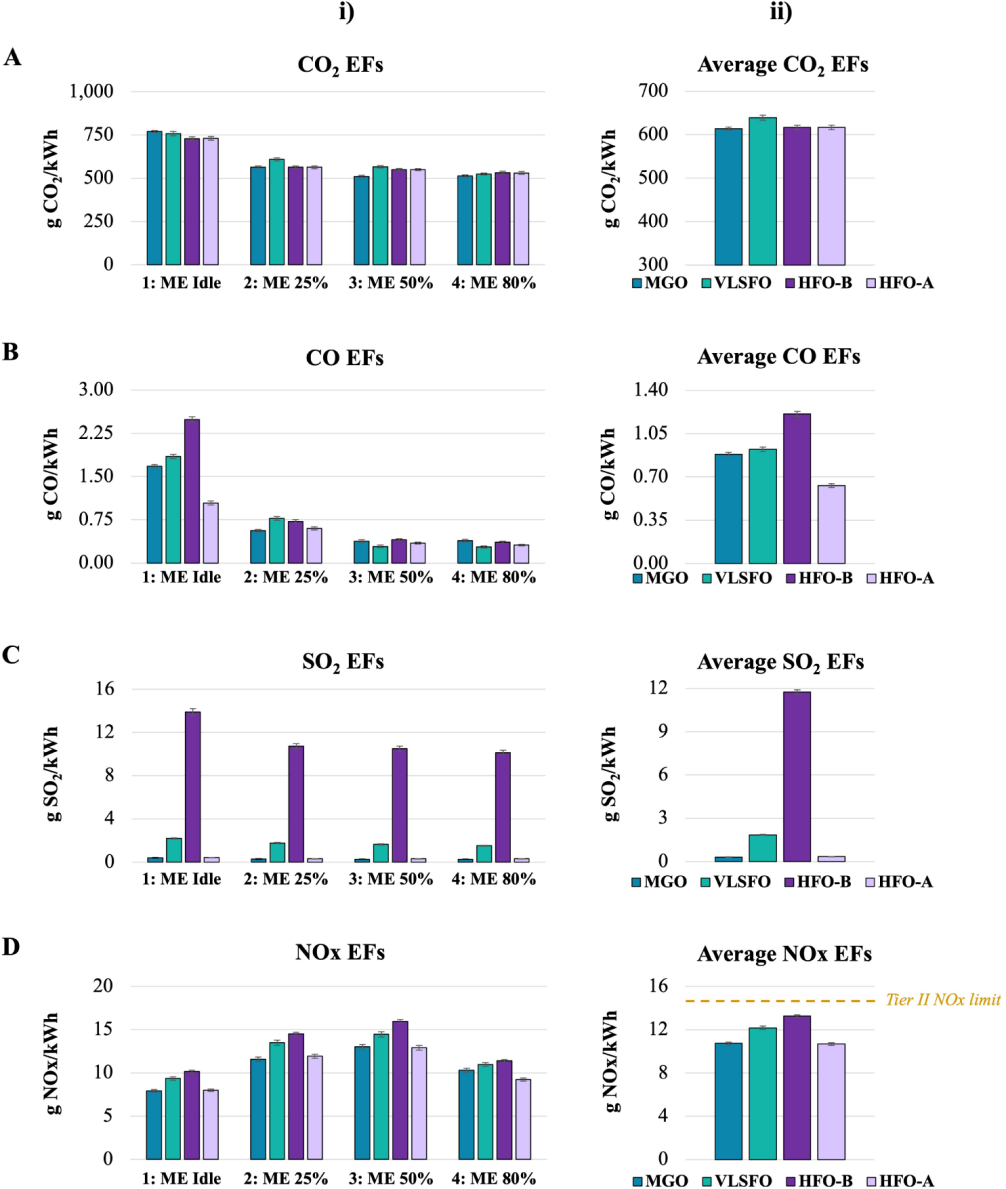
Gas EFs. Columns are ordered as follows: EFs per engine operating mode (i), vessel weighted average EFs (ii). (A) CO_2_ EFs. (B) CO EFs. (C) SO_2_ EFs. (D) NOx EFs; the orange dotted line represents the Tier II NOx emissions limit for bulk carriers operating with engine speed <130 rpm, i.e., 14.4 g kW^–1^ h^–1^. HFO-B and HFO-A stand for upstream (before) and downstream (after) of the scrubber, respectively.

The CO_2_ EFs are presented in Figure 2A(i). The same trend is observed across all fuels, with emissions about 20% higher in idle mode and gradually decreasing as ME loads increase. This is anticipated due to lower combustion efficiency of smaller displacement four-stroke engines at medium loads,^9,18^ and improved combustion efficiency at higher ME loads.^50^ No difference in CO_2_ emissions is observed upstream and downstream of the scrubber, while differences among fuels are minimal. During idle mode, MGO shows the highest absolute mean CO_2_ emissions, about 6% higher than HFO. In modes 2 and 3, VLSFO has slightly higher emissions. In mode 4, HFO emissions are 3% higher than MGO.

Figure 2A(ii) shows the weighted average CO_2_ EFs, with no difference for HFO upstream and downstream of the scrubber, or between HFO and MGO. VLSFO has a weighted average CO_2_ EF about 4% higher than MGO and HFO, as expected due to its higher carbon (C) content (Tables S2 and S3) and specific fuel consumption (SFC) (Table S5).^50,63^ Even though VLSFO is a blend of HFO and MGO, it often contains additional blend stocks and additives to meet sulfur regulations, enhance performance or improve handling properties. These components, such as cracked or aromatic hydrocarbons, can increase the aromatic or unsaturated hydrocarbon fraction, thereby raising its overall C content to levels exceeding those of both HFO and MGO. Similarly, its SFC can be affected. The weighted average EFs in g CO_2_/g fuel are 3.16 ± 0.03 for HFO, 3.23 ± 0.03 for VLSFO and 3.21 ± 0.02 for MGO. The measured EFs align well with the calculated EFs following IMO’s rationale.^50^

The measured CO_2_ emissions are representative of two-stroke, slow-speed diesel engines for similar fuels and engine modes.^9,16,18,19^ No difference on CO_2_ levels upstream and downstream of the scrubber has also been reported in the literature,^17,35,64^ indicating that the scrubber has no effect on CO_2_ concentrations, in agreement with our measurements.

##### 3.2.1.2 CO Emissions

A consistent trend is observed among the three fuels, with higher values during idle mode and a gradual decrease as ME loads increase (Figure 2B(i)). During idle mode, HFO exhibits its highest CO EF upstream of the scrubber, 2.49 ± 0.05 g/kW h, followed by a reduction of approximately 60% downstream of the scrubber. VLSFO shows about 10% higher CO emissions than MGO, and about 80% higher than HFO after the scrubber. CO values for all fuels are significantly lower in modes 2 to 4, fluctuating within the same ranges in each mode. Post-scrubber reductions ranging from 17% to 13% are observed, going from mode 2 to mode 4.

The weighted average CO emissions are reduced by ∼50% downstream of the scrubber, reaching 0.63 ± 0.02 g/kW h (Figure 2B(ii)). The weighted average post-scrubber CO EF of HFO is ∼ 30% lower than that of VLSFO and MGO, which are about 0.9 g/kW h.

Similar CO values have been reported in the literature.^16,19^ ICCT (2020)^35^ reports an 11% post-scrubber reduction in CO emissions. Conversely, Yang et al. (2021)^9^ found no CO reductions after the scrubber during ME operation. However, they observed substantially higher CO emissions while the ME was idle and noted a 53% reduction in CO post-scrubber emissions in this case, consistent with our findings. A small amount of solid residue accumulates on the scrubber walls over time and is periodically collected and properly disposed of onshore, as detailed in SI Note 13. Elemental analysis of this residue (Figure S21) revealed the presence of transition metals, including vanadium, nickel, and cobalt, among other elements. The observed reduction in CO concentrations downstream of the scrubber may be attributed to oxidation processes occurring within the scrubber. This oxidation could potentially be catalytic in nature and could be tentatively linked to the presence of trace amounts of these transition metals in the accumulated residue. However, the exact mechanism behind this phenomenon remains unclear and is beyond the scope of this study. Future studies would be needed to explore this potential catalytic oxidation effect, comparing these results with theoretical kinetic modeling or catalytic reaction mechanisms, while considering the scrubber residence time, volume, and inner surface area.

##### 3.2.1.3 SO_2_ Emissions

SO_2_ emissions are reduced by 97% downstream of the scrubber (Figure 2C(i)). SO_2_ EFs for all fuels are 25–30% higher during idle mode compared to when the ME is operating, with values within the same ranges across modes 2 to 4, i.e., ∼0.30 g/kW h for MGO and HFO after the scrubber, and ∼ 1.65 g/kW h for VLSFO. Regarding vessel’s weighted average EFs (Figure 2C(ii)), the post-scrubber SO_2_ emissions of HFO are very close to those of MGO (0.35 vs 0.31 ± 0.01 g/kW h), while being 80% lower than those of VLSFO (1.85 ± 0.03 g/kW h). SO_2_ emissions are fuel dependent; hence, these results are expected, given the S contents of the fuels and the operation of the scrubber.^50^ Similar SO_2_ EFs have been reported in the literature.^65^

##### 3.2.1.4 NOx Emissions

The same trend is observed for all fuels, with the lowest EFs during idle mode, a gradual increase up to mode 3, followed by a decrease in mode 4 (Figure 2D(i)). This can be attributed to NOx emissions increasing with higher combustion temperatures and improved combustion efficiency.^13^ An approximate 20% reduction in NOx emissions is observed downstream of the scrubber in all modes, rendering HFO post-scrubber emissions almost equal to those of MGO for modes 1–3, and 11% lower in mode 4. VLSFO has higher NOx emissions than MGO in all modes, ranging from 18% higher in idle to 6% higher in mode 4. Similarly, it has over 12% higher post-scrubber emissions than HFO in all modes.

The weighted average NOx EF for HFO is reduced by 20% downstream of the scrubber, being almost equal to MGO (∼11 g/kW h) and 12% lower than VLSFO (∼12 g/kW h) (Figure 2D(ii)). Although NOx emissions are not fuel but rather combustion dependent,^19,50^ the nitrogen (N) content and density of the fuel are correlated with NOx emissions, with higher values typically resulting in higher emissions.^59,63^ Therefore, these results can be partly explained by MGO having the lowest N content and density among the tested fuels, followed by VLSFO and HFO (Tables S2 and S3).

Previous studies report similar NOx emissions for the tested fuels.^18,66,67^ Yang et al. reported a 42% reduction in post-scrubber NOx emissions during ME idle conditions and while operating four-stroke diesel generators.^9^ However, unlike our findings, no significant reduction in NOx emissions downstream of the scrubber has been observed during ME operation.^9,35,52,66^ Although the mechanism behind our observed reduction in NOx post-scrubber emissions is unknown, it could potentially be attributed to several factors.

The reduction in NOx emissions likely reflects a reduction in NO, which has low solubility in seawater.^68^ NO if converted to NO_2_, can dissolve in seawater and be removed as nitrates. Yet, an oxidizing agent would be required for this, and although dissolved oxygen in seawater could contribute to such oxidation, this reaction is very slow. Moreover, our N mass balance calculations (Figure S17) show that the amount of N in scrubber washwater is much lower than expected if significant NO oxidation were occurring, reflecting less than 9% of gaseous NOx removal.

Another potential explanation for the observed NOx reduction could involve a selective catalytic reduction-like process, converting NOx into N_2_ and H_2_O.^68,69^ This may be mediated by amine groups found in organic molecules in seawater, which could undergo diazotization in the presence of the NOx and Cl ions.^70^ The resulting diazonium salts could be decomposed to N_2_ either thermally or catalytically, potentially facilitated by the presence of transition metals in the solid residue inside the scrubber walls. However, this hypothesis remains speculative, and further experiments are needed to elucidate the specific mechanisms at play, including catalytic testing of the scrubber residue and measurement of additional N species and volatile organic compounds upstream and downstream of the scrubber.

#### 3.2.2 PM_2.5_ Emissions

**Figure 3:**
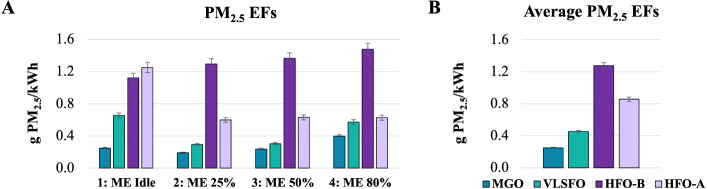
PM_2.5_ EFs. (A) EFs per engine operating mode. (B) Vessel weighted average EFs. HFO-B and HFO-A stand for upstream (before) and downstream (after) of the scrubber, respectively.

PM_2.5_ EFs show increased values during idle mode, which decrease while the ME is operating at 25% load, followed by a slight gradual increase with increasing ME loads (Figure 3A). During idle mode, a 11% increase in the absolute mean PM_2.5_ emissions is observed downstream of the scrubber, while in modes 2–4, post-scrubber PM_2.5_ emissions are reduced by over 54%. In idle mode, PM_2.5_ emissions after the scrubber are about double those of VLSFO and five times higher than those of MGO. In modes 2 and 3, PM_2.5_ emissions downstream of the scrubber remain about twice as high compared to VLSFO and about three times higher than MGO. These differences are significantly lowered in mode 4, where post-scrubber PM_2.5_ emissions are almost equal to VLSFO and approximately 60% higher than MGO. The weighted average EFs across all modes (Figure 3B) show about 30% reduction in PM_2.5_ emissions downstream of the scrubber (from 1.28 ± 0.04 to 0.86 ± 0.03 g/kW h), being almost twice as high compared to VLSFO (0.45 ± 0.01 g/kW h) and over three times higher than MGO (0.25 ± 0.01 g/kW h).

PM_2.5_ emissions are linked to the fuels’ S content.^50^ Therefore, the observed trend can be explained by the respective S contents (Tables S2 and S3). Using IMO’s methodology^50^ to estimate PM_2.5_ EFs, the calculated values align closely with the measured weighted averages during ME operation: ∼0.2 g/kW h for MGO, ∼0.3 g/kW h for VLSFO, and ∼1.4 g/kW h for HFO upstream of the scrubber. PM_2.5_ EFs are in the same value ranges with those reported in the literature.^9,16,18,19,65,66^

Similar post-scrubber PM_2.5_ reductions have been reported in the literature during ME operation.^2,66^ However, some studies report no reduction in PM_2.5_ emissions downstream of the scrubber,^52^ while others observe either increased post-scrubber PM_2.5_ emissions^64^ or substantial reductions of about 75% for similar ME loads.^71^ PM_2.5_ measurements are greatly affected by the adopted methodology,^9,53,54^ even when following the ISO 8178 guidelines.^30^ In scrubber environments, PM_2.5_ measurements are even more challenging due to the presence of both solid and condensable particles. Sudden temperature drops can result in high post-scrubber PM_2.5_ values due to condensation of volatile and semivolatile organic compounds. The condensation of water vapors that might be present downstream of the scrubber can also inflate PM_2.5_ measurements.^72^ On the contrary, high dilution ratios and elevated temperatures, can overestimate the scrubber’s PM_2.5_ removal by causing condensable components, including sulfuric acid particles and organic PM, to revert to the gas phase.^9,53^ Differences in engine types and measurement setups can also greatly affect measurements, complicating the generalization of PM_2.5_ reduction through scrubbers or comparisons between studies.

Few studies have reported PM_2.5_ measurements at idle conditions. For instance, Yang et al. (2021)^9^ observed a 38% reduction in post-scrubber emissions at such conditions. This contrasts with our measurements, which show a slight increase in PM_2.5_ post-scrubber during idle mode. Although it is well-known that PM_2.5_ emissions depend on the fuel S content, particulate formation is a complex process not yet fully understood.^73^ The observed increase in PM_2.5_ post-scrubber may be due to several factors. During idle mode, the water flow rate through the scrubber is lower, resulting in fewer particles being scrubbed. The lower combustion temperatures in this mode could also facilitate potential nucleation effects. Additionally, there might be a connection to the solid residue present on the scrubber walls (SI Note 13), which could be releasing particles during this mode. Measuring the number of particles and particle fractions could help shed light on this effect.

#### 3.2.3 Washwater Emissions

The pH of washwater decreases as ME loads increase (Table 1), as expected, due to higher fuel consumption and exhaust gas flow rates at higher loads. The lowest pH observed is 3.99. According to the IMO,^14^ the washwater pH should not fall below 6.5 at a distance of 4 m from the overboard discharge point when the ship is stationary. The lowest discharge pH measured is well above 3, which has been identified as the critical value to meet the IMO limit, according to Oldendorff’s data and Japan’s Ministry of Land, Infrastructure, Transport and Tourism (MLIT).^22^

**Table 1:** pH and Turbidity of Incoming Seawater (Inlet) and Discharged Washwater (Outlet), and Discharge Flow Rates in Different Engine Modes

Quality parameter	Mode 1: ME idle		Mode 2: ME ∼ 25%		Mode 3: ME ∼ 50%		Mode 4: ME ∼ 80%	
	Inlet	Outlet	Inlet	Outlet	Inlet	Outlet	Inlet	Outlet
pH^a^	8.10	5.44	8.09	5.18	8.40	4.13	8.15	3.99
Turbidity (FNU)	0.60 ± 0.01	0.53 ± 0.01	0.43 ± 0.01	1.86 ± 0.04	0.80 ± 0.02	2.18 ± 0.04	0.52 ± 0.01	2.49 ± 0.05
Discharge flow rate (t/MW h)	254		73		68		48	

a Measurement error: ±0.01 in all readings.

In contrast to pH, turbidity values in the washwater increase with increasing engine loads (Table 1). Observed values are well below the IMO threshold, which limits continuous discharge turbidity to 25 FNU above the inlet.^14^

##### 3.2.3.1 Nutrients

**Figure 4:**
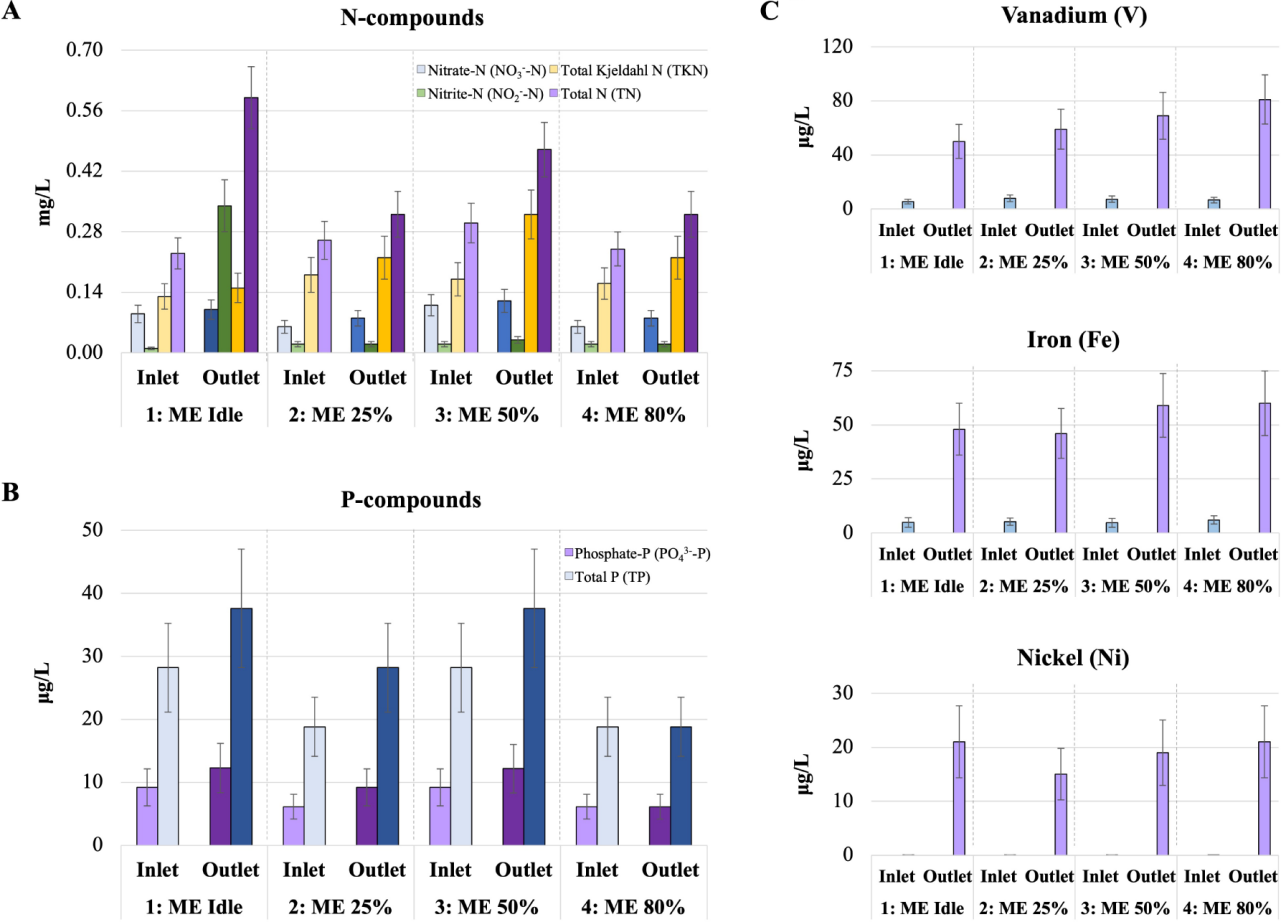
Nutrients and selected metals in seawater (inlet) and scrubber washwater (outlet) per engine mode. (A) N-compounds; (B) P-compounds; lighter colors indicate concentrations in incoming seawater and darker colors in washwater. (C) V, Fe, and N concentrations.

Total N is increased in the washwater in modes 1 and 3, primarily due to nitrates and Kjeldahl N, respectively (Figure 4A). For phosphates and total P, although the absolute mean concentrations are increased in the washwater in modes 1–3, no statistically significant differences can be observed between inlet and outlet at the parts per billion scale of measurements (Figure 4B).

IMO^14^ limits nitrate discharge to the equivalent of a 12% removal of NOx from exhaust or 60 mg/L normalized for a washwater discharge rate of 45 tons/MW h, whichever is greater, to prevent eutrophication of coastal waters. As discussed in Section 3.2.1.4, the measured nitrates in washwater reflect less than 9% NOx removal from the exhaust (Figure S17). Additionally, the relevant concentration thresholds, given the discharge flow rates in each mode (Table 1), would be 11, 37, 39, and 48 mg/L for modes 1–4, respectively. In all modes total N is below 1 mg/L, hence, far below the IMO limits. No limits are suggested for P concentrations, as N is the limiting nutrient for eutrophication in marine environments.

To contextualize scrubber’s N and P emissions, we compared them to the discharge requirements for urban wastewater treatment plants (WWTPs) in European Union (EU) and the US. Given an average washwater discharge of 10 million L/day, the scrubber can be considered equivalent to a WWTP serving 50,000 people. The relevant EU discharge limits for sensitive water bodies are 15 mg/L for total N and 2 mg/L for total P,^74,75^ with proposed stricter limits of 10 mg/L for total N and 0.7 mg/L for total P.^76^ Similarly, US EPA^77^ limits total N to 10 mg/L and total P to 1 mg/L. These limits can vary for specific local conditions, with the strictest being 3 mg/L for total N and 0.1 mg/L for total P. The observed N and P concentrations are well below even the strictest limits.

##### 3.2.3.2 Metals and Total Suspended Solids (TSS)

Out of the 23 metals tested (Table S6), only 10 showed statistically significant increases in the scrubber washwater: aluminum (Al), chromium (Cr), cobalt (Co), copper (Cu), iron (Fe), lead (Pb), mercury (Hg), nickel (Ni), vanadium (V), and zinc (Zn) (Figures 4C and S23). The highest levels observed for V, Fe, Ni, and Cu, which is primarily due to HFO composition (Table S3).

Currently, there are no limits for metals in scrubber washwater. To put the observed metal concentrations into perspective, we compared them with strict US^78,79^ and EU^80^ limits for industrial facilities treating a wide range of effluents (Table S7; SI Note 13). Moreover, as scrubber washwater is expected to dilute several hundred thousand times after being discharged into the ocean,^81^ we assumed a conservative 1000-fold dilution of the maximum observed concentrations, and compared the expected final concentrations of scrubber pollutants in receiving water bodies with the EU environmental quality standards (EQS) for priority pollutants,^82−85^ and the US EPA water quality criteria for aquatic life in seawater^86,87^ (Table S7; SI Note 13). Both the measured maximum metal concentrations in scrubber washwater and the 1000-fold diluted concentrations are orders of magnitude below the relevant industrial wastewater discharge limits and environmental criteria, respectively.

TSS concentrations increased at the outlet in modes 1–3 (Table 2). TSS in washwater is not regulated, hence, we compared the measured values with limits from US^78^ and EU^80^ industrial wastewater regulations to contextualize these values, similar to our approach with metals. As shown in Table 2, the observed values are below these limits. However, considering only the outlet concentrations might not be appropriate, as higher TSS outlet values correlate with higher inlet ones, indicating that incoming seawater quality affects TSS levels in scrubber effluents. Factors like sediment resuspension, organic matter influx, and anthropogenic pollution contribute to variations in seawater TSS levels.^88,89^ Therefore, the observed TSS washwater values reflect baseline seawater conditions as well, rather than just scrubber operation.

**Table 2:** TSS Measurements per Mode and Comparison to US and EU Industrial Discharge Limits

TSS concentrations (mg/L)								Max TSS limits for industrial wastewater (mg/L)
Mode 1: ME idle		Mode 2: ME 25%		Mode 3: ME 50%		Mode 4: ME 80%		
Inlet	Outlet	Inlet	Outlet	Inlet	Outlet	Inlet	Outlet	
22 ± 1.8	40 ± 3.2	34 ± 2.7	46 ± 3.7	24 ± 1.9	56 ± 4.5	36 ± 2.9	40 ± 3.2	74.1;^78^ 100^80^

##### 3.2.3.3 Organic Compounds

PAHs were not detected in the inlet seawater across the four engine modes or in the scrubber discharge of mode 1. In mode 2, fluoranthene, fluorene, and phenanthrene were detected, 0.01 ± 0.008, 0.05 ± 0.03, and 0.25 ± 0.11 μm/L respectively, while only phenanthrene was detected in modes 3 (0.13 ± 0.08 μm/L) and 4 (0.05 ± 0.03 μm/L). According to the IMO,^14^ the maximum continuous PAH concentrations in scrubber washwater should not exceed 50 μm/L phenanthrene equivalents above the inlet water PAH concentrations, normalized for a discharge flow rate of 45 t/MW h. The relevant concentration thresholds, given the discharge flow rates in each mode (Table 1), would be 9, 31, 33, and 47 μm/L for modes 1–4, respectively. Although the method for calculating total PAH concentrations in phenanthrene equivalents is not specified by the IMO, simply adding the observed concentrations yields total PAH levels of 0, 0.31 ± 0.12, 0.13 ± 0.08, and 0.05 ± 0.03 μm/L for modes 1–4, respectively, all well below the relevant IMO thresholds. These results align with PM_2.5_ measurements, confirming a potential connection between PM removal from scrubbers and presence of PAHs in washwater,^27^ since no PAHs were detected during the idle mode where no PM_2.5_ removal was observed.

BTEX compounds were not detected in the incoming seawater for any mode or in the outlet for mode 1. Only toluene was detected in scrubber washwater at the following concentrations: 7.6 ± 3.4 μm/L in mode 2, 5.6 ± 2.5 μm/L in mode 3, and 1.3 ± 0.6 μm/L in mode 4. Additionally, 1,4-dichlorobenzene was not detected in either the inlet or outlet for any mode.

Oil and grease concentrations were found to be below 1 mg/L in both the inlet and outlet for all modes. The hydrocarbon oil index was similar for inlet and outlet for mode 1, with values of 3 ± 1 μm/L and 4 ± 2 μm/L, respectively. However, it increased significantly downstream of the scrubber for the rest of the modes, rising from 3 ± 1 to 40 ± 12 μm/L in mode 2, from 5 ± 2 to 50 ± 15 μm/L in mode 3, and from 3 ± 1 to 30 ± 9 μm/L in mode 4.

Concentrations of BTEX compounds, oil, grease, and hydrocarbons are not regulated by the IMO. To put the measured values into perspective, we compared them with some of the strictest maximum daily US^90,91^ and EU^80^ limits for industrial wastewater discharge (Table S8, SI Note 13). Moreover, assuming a conservative 1000-fold dilution in open sea, we compared the expected final concentrations in receiving water bodies with the EU EQS for priority pollutants^84^ and the US EPA water quality criteria for aquatic life^86^ (Table S8, SI Note 13). Both the measured maximum concentrations in scrubber washwater and the diluted concentrations are below the relevant limits and environmental criteria, respectively.

##### 3.2.3.4 Comparison with Prior Studies on Scrubber Washwater and Potential Impacts

The measured inlet concentrations were well within native ranges,^92,93^ suggesting that the vessel’s equipment did not introduce contamination to the incoming seawater. In addition, measured outlet concentrations for most pollutants were within the ranges reported for open-loop scrubbers, e.g., refs (20,23,24,94−96). However, some studies^20,23,24,94,96^ are reporting significantly higher BTEX, total hydrocarbon and PAH concentrations, but inherent study limitations, such as high inlet concentrations or lack of incoming seawater concentrations, do not allow for meaningful comparisons. A detailed comparison of the measured concentrations of the above-discussed pollutants in scrubber washwater with prior studies is provided in SI Note 14.

Metals and PAHs can greatly affect aquatic ecosystems if their concentrations exceed certain thresholds.^86,97^ To assess the impacts of scrubber washwater on aquatic ecosystems we need to factor in the expected dilution and dispersion of these discharges, both immediately after their release as well as over longer time periods. Although actual dilution rates depend on various factors, including currents, wave action, and discharge rates, effluents from ocean-going vessels are expected to be diluted by tens to hundreds of thousands within the first minutes of discharge, and potentially by millions over time and distance.^22,81^

Previous ecotoxicity assessments of open-loop scrubber washwater have reported statistically significant effects on aquatic organisms at concentrations ranging from a 100-fold dilution to no dilution.^20,23,24,96,98,99^ Jalkanen et al. (2024)^23^ reported no effect concentrations after diluting effluents 100,000 times for the most sensitive end points analyzed, with the exception of green sea urchin larvae, where no effect concentrations would require higher than 1,000,000-fold dilutions.

However, given that these effects were observed over several hours or even days of exposure, and the expected dilution factors in practice are much higher,^81,100^ while aquatic species can move between various streams,^27^ the reported adverse effects on aquatic organisms are unlikely in open-sea environments, as verified by several prior modeling studies.^22,53,95,96,101,102^ This may not be the case in confined spaces with higher traffic and lower water exchange rates, such as ports, where the dilution and dispersion of the released compounds are limited.^53,94^ In such areas, the discharge of scrubber washwater might lead to localized accumulation of pollutants, thereby increasing the risk of adverse impacts on aquatic ecosystems particularly in sensitive regions.^103^ Therefore, additional experimental data on scrubber discharges over longer periods and under various conditions are needed to assess their short- and long-term whole effluent toxicity, under realistic dilutions, following reliable risk assessment methodologies.^27,55^

### 3.3 Well-To-Wake (WtW) Assessment

**Figure 5:**
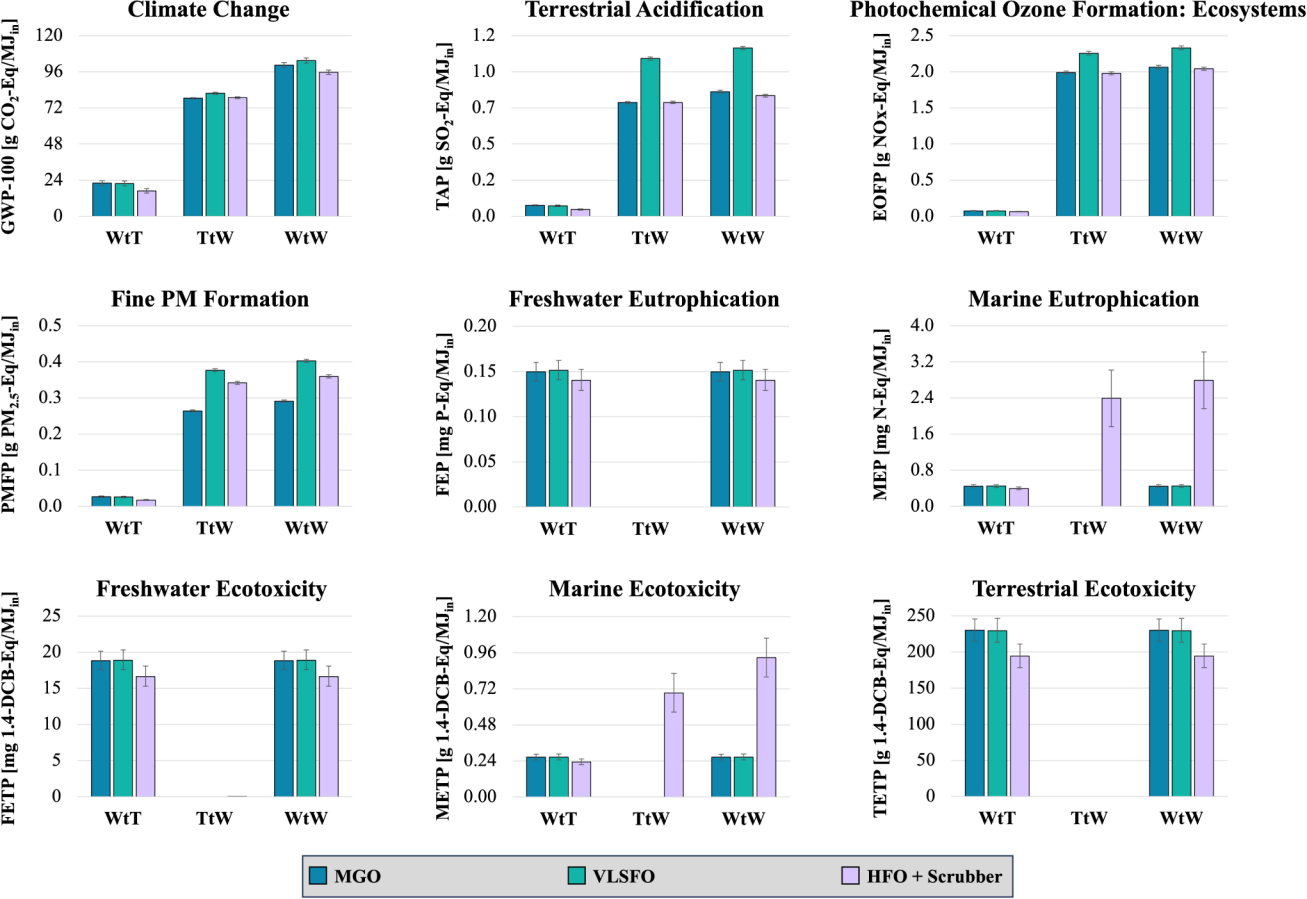
WtW Impacts of MGO, VLSFO and HFO combined with a scrubber per MJ of incoming energy into the vessel’s engines. GWP-100: global warming potential over a 100-year time horizon; TAP: terrestrial acidification potential; EOFP: ecosystem ozone formation potential; PMFP: particulate matter formation potential; FEP: freshwater eutrophication potential; MEP: marine eutrophication potential; FETP: freshwater ecotoxicity potential; METP: marine ecotoxicity potential; TETP: terrestrial ecotoxicity potential; 1,4-DCB: 1,4-dichlorobenzene. For HFO + scrubber post-scrubber values were considered.

We notice the same trend for climate change, terrestrial acidification and photochemical ozone formation, with HFO and scrubber having equal or lower WtW impacts compared to low-S fuels (Figure 5). More specifically, HFO and scrubber has 5% lower GHG WtW emissions than MGO, and 8% lower than VLSFO. HFO has similar SO_2_-Eq WtW emissions with MGO, being ∼30% lower than VLSFO. Similarly, HFO and scrubber has similar photochemical ozone formation impacts with MGO, being ∼10% lower than VLSFO. Similar results hold true for human health ozone formation (Figure S24).

Regarding fine PM formation, post-scrubber HFO impacts are 24% higher than MGO and ∼10% lower than VLSFO. This is anticipated given HFO’s higher PM_2.5_ emissions during combustion compared to low-S fuels, but lower SO_2_ and NOx combustion emissions compared to VLSFO. SO_2_ and NOx emissions are considered for the assessment of this indicator as they contribute to the formation of secondary PM_2.5_ aerosols.^44^ To make post-scrubber HFO PM formation impacts equivalent to MGO, the adoption of PM abatement options would be necessary. Effective PM abatement technologies suitable for diesel engines and high-S fuels have demonstrated substantial PM emission reductions, achieving levels significantly lower than those of MGO.^2,3,52^

Freshwater eutrophication concerns impacts from either direct releases or potential transfer of P and phosphates from soil to freshwater bodies.^44^ Relevant TtW emissions to seawater do not contribute to freshwater eutrophication. Therefore, the WtW impacts on freshwater eutrophication are equal to the WtT ones, which are almost equivalent among the three systems. The same applies to WtW freshwater and terrestrial ecotoxicity. The impact of relevant scrubber washwater releases on freshwater and terrestrial systems is considered negligible, with characterization factors close to zero.^44^ Although WtW freshwater ecotoxicity impacts are similar for the three analyzed systems, HFO with scrubber has approximately 15% lower impacts in the case of terrestrial ecotoxicity.

The calculated marine eutrophication and ecotoxicity WtW impacts of HFO with scrubber are about 6 and 3 times higher than those of low-S fuels, respectively. However, these results should be interpreted with caution. Although in absolute values, these indicators show a greater potential for marine eutrophication and ecotoxicity in the case of HFO with a scrubber, adverse impacts on receiving marine ecosystems are unlikely from ocean-going vessels in open seas, as discussed in Section 3.2.3.4. Moreover, the characterization factors^44,104^ used to calculate these impacts do not differentiate between stationary and moving pollution sources. This poses challenges if the analyzed pollution source is a moving vessel, as the dispersion and dilution patterns differ significantly from those of stationary sources, such as WWTPs, due to the turbulence caused by propellers and hull displacement.

Scrubber washwater is diluted tens to hundreds of thousands of times immediately after discharge.^81^ Assuming a conservative initial dilution factor of 10, marine eutrophication and ecotoxicity TtW impacts get lower than the WtT ones (from 2.4 ± 0.6 mg N-Eq/MJ_in_ to 0.24 ± 0.06 mg N-Eq/MJ_in_, and from 0.7 ± 0.1 g 1,4-dichloronenzene-Eq/MJ_in_ to 0.07 ± 0.01 g 1,4-dichloronenzene-Eq/MJ_in_, respectively), leading to similar WtW impacts among the three fuels. This example underscores the limitations of current LCA methodologies in accurately assessing impacts on marine ecosystems, emphasizing the need for revised characterization models. There is no widely accepted marine eutrophication and ecotoxicity characterization model, and existing methodologies involve characterization factors of high uncertainty, even for stationary pollution sources.^105,106^ Therefore, the relevant results in Figure 5 are reported only for discussion purposes, and cannot be used to conclude that HFO with scrubber has greater eutrophication or ecotoxicity impacts compared to low-S fuels.

Limited WtW LCA studies exist on the considered fuels, focusing mostly on climate change and acidification impacts, and often not being informed by on-board measurements under similar conditions. A detailed comparison with prior studies is provided in SI Note 15.

Considering all the LCA impact categories, it can be concluded that, if PM abatement options are adopted, HFO with a scrubber can be considered equal to the use of MGO, while outperforming VLSFO in several impact categories, for large, ocean-going bulk carrier vessels in open seas. From an industry perspective, VLSFO is the most relevant fuel to compare with HFO and a scrubber. However, both VLSFO and MGO are included here for the sake of experimental rigor and scientific interest.

Overall, this work challenges the notion that end-of-pipe solutions outperform start-of-pipe ones, emphasizing the need for holistic LCA studies. The adoption of such cradle-to-grave approaches, supported by robust data and accounting for various impacts, can enable effective assessments of different fuel systems, pollution abatement and decarbonization technologies, avoiding perverse incentives, and expediting maritime decarbonization.

## Data Availability

Excel files with all the collected data and conducted calculations are available upon request, free of charge.

## Supporting Information Available

Supporting Notes 1–15: LCA Methodology and Data: Notes 1 and 2 outline the LCA framework for the fuels and the scrubber. LCA TtW Impacts and Functional Unit Selection: Notes 3 and 4 explain TtW impact indicator calculations and the rationale for selecting the study’s functional unit. Vessel, Engines, Scrubber and Fuels: Note 5 details the vessel’s technical specifications, while Note 6 presents chemical analyses of fuels, cylinder oils, and lubricants. Operating Conditions and Emission Measurements: Notes 7 and 8 describe engine/scrubber operating modes and emission measurements, respectively. Seawater and Washwater Analyses: Notes 9 and 10 cover sampling methods, preservation, and analytical procedures. Emission Factors and LCA Results: Note 11 explains EF calculations, while Notes 12–15 present WtT, TtW, and WtW LCA results, including comparisons with prior studies. Supporting Tables S1–S8: Scrubber Materials and Fuel Chemistry: Table S1 lists scrubber construction materials, while Tables S2–S4 provide fuel and lubricant analyses. Engine Conditions and Washwater Analyses: Table S5 outlines engine operating modes; Table S6 lists seawater and washwater parameters. Regulatory Comparisons for Scrubber Washwater: Tables S7 and S8 compare washwater pollutant concentrations to U.S. & EU wastewater discharge regulations and environmental criteria. Supporting Figures S1–S24: Scrubber System and Fuels: Figures S1–S3 depict transportation of scrubber materials and parts, scrubber components, system layout, and vessel propulsion. Figures S4–S7 show fuel analyses before bunkering. Instrumentation, Sampling Setup and Measurement Locations: Figures S8–S14 illustrate CEMS analyzers, dilution/sampling setups, and water quality monitoring equipment. LCA and Mass Balance Results: Figures S15, S16, and S24 present WtT and WtW impacts of fuels and scrubber, while Figure S17–S19 detail N, S, and C mass balance during scrubber operation. Scrubber Residue and Water Quality: Figures S20–S24 include scrubber residue images, residue elemental analyses, and metal concentrations in seawater and washwater (PDF)
