# Correction to “Marine Scrubbers vs Low-Sulfur Fuels: A Comprehensive Well-To-Wake Life Cycle Assessment Supported by Measurements Aboard an Ocean-Going Vessel”

**DOI:** 10.1021/acs.est.6c06564

**Published:** 2026-07-06

**Authors:** Patritsia M. Stathatou, Ievgenii Petrunia, Torsten Barenthin, George Gotsis, Paul Jeffrey, Christopher Fee, Scott Bergeron, Marios Tsezos, Michael Triantafyllou, Neil Gershenfeld

In section 3.2.3.3 (Organic Compounds), all concentrations reported in μm/L should be corrected to μg/L.

