# Supplementary material for: Marine Scrubbers vs Low-Sulfur Fuels: A Comprehensive Well-To-Wake Life Cycle Assessment Supported by Measurements Aboard an Ocean-Going Vessel
Source: Environ Sci Technol. 2025 Apr 4;59(14):7066–80. doi: 10.1021/acs.est.4c10006 (PMC12004910; doi:10.1021/acs.est.4c10006)
Supplement: Supplementary file 1 — es4c10006_si_001.pdf [file es4c10006_si_001.pdf]

Supporting Information

Marine scrubbers vs. low-sulfur fuels: A comprehensive well-to-wake life cycle assessment supported by measurements aboard an ocean-going vessel

Patritsia M. Stathatou, Ievgenii Petrunia, Torsten Barenthin, George Gotsis, Paul Jeffrey, Christopher Fee, Scott Bergeron, Marios Tsezos, Michael Triantafyllou, and Neil Gershenfeld*

This file consists of 57 pages (S1-S57) and includes the following supporting information providing detailed description of the employed methodology and data used, the vessel's characteristics, the onboard measurements and the LCA results:

Supporting Notes 1-15

Supporting Tables S1-8

Supporting Figures S1-S24

References

Note 1 | LCA Methodology & Data Used - Fuels

The average global impacts linked to the production and distribution of HFO and MGO were calculated, using the ecoinvent v3.10 database[1]. Specifically, for HFO, we selected the market activity for heavy fuel oil (HFO), Rest of World (RoW), using the cut-off system model. For MGO, we selected the market activity for diesel production, low-sulfur, RoW, also using the cut-off system model.

MGO (sulfur content: <0.1%) consists exclusively of distillates and is considered similar to low sulfur diesel in terms of refining processes involved (crude oil fractional distillation, desulfurization and further refining), calorific value, sulfur content and intended use[2]. In fact, in some areas of the world, MGO (Distillate Marine Fuel - DMA) is indeed automotive diesel (not including biodiesel), with added marker and dye and without the excise duty applicable to automotive fuel[3]. Therefore, the ecoinvent data for low sulfur diesel were used as proxy for MGO. Ecoinvent data are provided per kg of fuel. We converted these data to the functional unit or the study, i.e., MJ of energy coming into the vessel's engines, given the calorific value of each fuel (Tables S2 and S3). Market ecoinvent activities have been considered for the WtT calculations to account for the impacts across the entire marine fuel supply chain.

For VLSFO's WtT impacts, not included in ecoinvent, a weighted average assumption of 15% HFO and 85% MGO was considered[4], approximating the blend ratio required to meet the IMO 2020 S limit (≤0.50%)[5]. Error values accounted for uncertainty of WtT impacts due to variations in fuel calorific values[6,7].

Note 2 | LCA Methodology & Data Used - Scrubber

Data were provided by the scrubber manufacturer, *i.e.,* Yara Marine Technologies. The main materials used for the tower included high-alloyed stainless steel, iron-nickel-chromium alloy, stone wool, and unalloyed steel. Reinforced steel and aluminum alloys were used for the exhaust gas damper, while cast iron, stainless steel, and aluminum were used for the pumps. PVC and glass fiber reinforced plastic composites (GFRP) were used for pipes and fitting. The total weight of materials considered for scrubber production was approximately 41,120 kg. The main materials and amounts (weight) used for the open-loop scrubber are presented in Table S1.

Most scrubber materials were sourced within Europe (e.g., from Germany, France, Sweden and Spain), while some motors and steel parts were produced in China. Sub-parts were either manufactured and assembled in Europe or were gathered in Gothenburg, Sweden, where the headquarters of the scrubber manufacturer are, and from there they were sent to China for manufacturing and assembly (Figure S1). Transportation of scrubber parts within China from manufacturing facilities to the shipyard in Beihai was also considered.

Table S1. Main materials used for scrubber production.

No	Component	Qty	Total weight [ton]	Materials	Notes
1	Scrubber Tower	1	1.23<br>5.17<br>1.86<br>3.29<br><br>1.65<br><br>0.36<br>0.88	EN 1.4404<br>EN 1.4529 (Alloy 926)<br>EN 1.4547 (254 SMO)<br>EN 2.4605 (Alloy 59)<br><br>Insulation (Wired mat Marine Insulation, t=100,0 mm)<br>S355<br>Steel, Galvanized	Materials for complete tower including suspension mounts, fixed points and demister
2	Exhaust gas damper	6	1.64<br>0.29	CorTen<br>Al-alloy	Damper body<br>Actuator
3	Process pumps	3	1.26<br>2.76<br>0.05<br>0.05	Super duplex (pump house & impeller)<br>Cast iron (motor)<br>Aluminium (demister pump motor)<br>316-L (demister pump house & impeller)	
4	Valves	32	0.77<br>0.1<br>0.002	Cast iron<br>Super duplex<br>PVC-U	Most vales have a body in cast iron and a liner to protect from corrosive media

The transportation steps of the various scrubber materials and parts is presented in Figure S1. For tower manufacturing, sheet plate steel materials were sourced from steel mill in Germany and transferred to Gothenburg, Sweden (Yara's location) via trucks. Steel sheets were then packed in pellets and sent to China via sea transport. The tower was manufactured in China; steel plate sheets were cut, rolled, welded and treated to be passivated. The tower was then packed and transferred via trucks to the shipyard (again in China) to be installed onto the ship. All auxiliary equipment (exhaust gas dampers, pumps, etc.) was sourced from across Europe and sent to Gothenburg, Sweden (Yara's location) via trucks. Then it was packed in containers and transferred to the shipyard in China, via sea transport, to be installed onto the ship.

To account for the impacts of the manufacturing and assembly, the energy consumed during these stages was considered and the impacts associated with the production of the medium voltage electricity needed in each case were assessed, taking into account the local energy mix. An estimated value of 2 MJ per kilogram of the weight of the relevant scrubber materials or parts was applied, as suggested in relevant literature[8,9]. The energy consumed for the installation of the scrubber onboard the vessel was not considered, as relevant impacts were expected to be minimal[9,10].

The ecoinvent v3.10 database[1] was used for calculating the scrubber WtT impacts. Scrubber end-of-life impacts, including disposal to landfill or incineration, were excluded from the analysis, as the scrubber manufacturer indicated that most of scrubber materials are recycled.

Two WtT assessments were conducted for the scrubber, using the functional units of a) one scrubber produced, and b) one MJ of incoming engine energy over the scrubber's 20-year lifetime (MJ_{in}). The incoming engine energy across the scrubber’s lifetime was calculated based on an average annual HFO consumption of ~7,830 tons (data from Oldendorff) and HFO's net specific energy (Table S3).

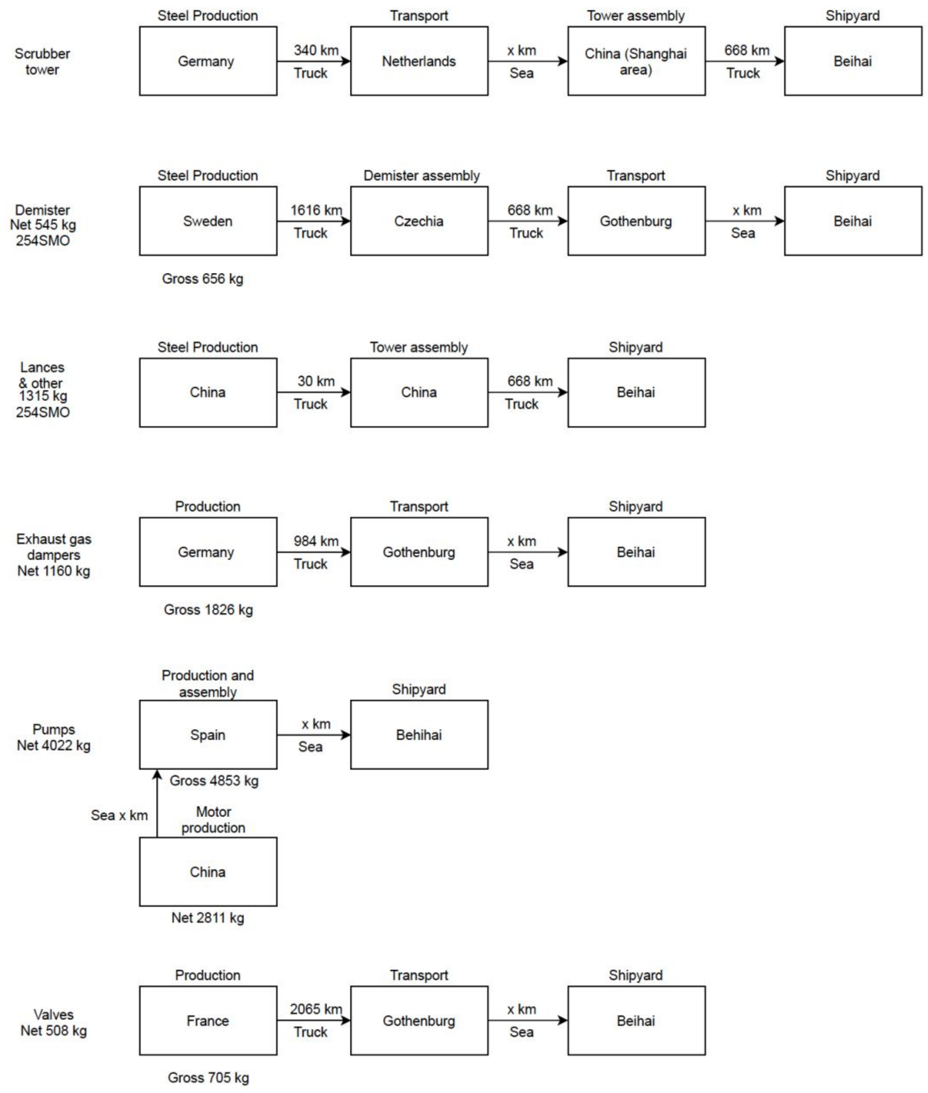

Figure S 1: Transportation of scrubber materials & parts.

Note 3 │ LCA – Calculation of TtW Impact Indicators

The weighted average gas and $PM_{2.5}$ EFs were converted to grams per MJ of energy input to the vessels' engines (g/MJ_{in}) considering an average engine efficiency of ~ 0.46[11], while the weighted average net releases of nutrients, heavy metals, and organic compounds reported in Section 3.2.3 were converted to g/MJ_{in} considering the discharge rates and the relevant engine outputs in each mode. TtW impact indicators were assessed following the ReCiPe, 2016 methodology[12].

Only the measured CO_2 emissions were considered for assessing the climate change TtW impacts. CH_4 and N_2O emissions in the TtW phase were not considered, since they weren't measured as they are typically very low to negligible in modern diesel engines[13,14].

Similarly, only SO_2, and NOx and $PM_{2.5}$ emissions were considered for assessing the terrestrial acidification and fine PM formation TtW impacts, since NH_3 emissions to air are negligible in modern diesel engines[13] and were not measured in the TtW phase. In addition, to assess the TtW photochemical ozone formation, only NOx emissions were considered.

For assessing the eutrophication and ecotoxicity TtW impacts, only the net measured emissions to seawater from the scrubber washwater were considered.

Note 4 | LCA – Functional Unit

The functional unit of the analysis is one MJ of energy input to the vessel engines (MJ_{in}). This functional unit was chosen to ensure consistency across all aspects of the analysis, including WtT and TtW phases, and to accurately account for the additional energy required by the scrubber.

The scrubber requires ~80–100 kW of power, which leads to increased fuel consumption during its operation. In our analysis:

- The calculation of the TtW emission factors accounts for respective fuel consumption in each case (including the additional fuel consumption to operate the scrubber in case of HFO), as well as the generated engine output from both the main engine (driving the propeller) and the auxiliary engines (supporting scrubber operation in case of HFO). This methodology is detailed in SI Note 10. This approach ensures that the energy required to operate the scrubber is fully incorporated into the analysis.

- To enable comparison with WtT values, the HFO TtW emission factors, originally expressed in g/kWh of engine output, were converted to g/MJin using the average engine efficiency (data provided by Oldendorff). This ensures consistency in the functional unit across the analysis, allowing for direct integration and comparison of WtT and TtW impacts.

Using a functional unit such as "1 MWh of work delivered to the propeller" or "1 ton of cargo transported over 1 km," while relevant in transport studies, would not be ideal for this specific study. This is because:

- The propeller is directly driven by the main engine, while the scrubber operation relies on auxiliary engines. Incorporating the scrubber's energy requirements into a functional unit tied to the propeller would create inconsistency, as the auxiliary engine energy requirements are not directly related to propulsion, and it could lead to confusion in the interpretation of the results.

- The current functional unit (MJin) provides a consistent basis for comparing impacts across all fuels and scrubber configurations, while ensuring that both propulsion and scrubber energy requirements are accurately captured.

Note 5 | Vessel, Engines & Scrubber Details

The Hedwig Oldendorff (IMO no. 9742728), built in 2018 and sailing under the flag of Portugal, has a deadweight capacity of 209,331 tons, length overall of 299.95 meters and width of 50.00 meters. The vessel's average speed is 10.40 knots, with a maximum of 16.00 knots. It is equipped with a MAN B&W 6G70ME-C9.2 slow-speed, two-stroke diesel main engine (ME), compliant with the IMO Tier II emission regulations for NOx emissions. The ME has a nominal maximum continuous rating (MCR) of 15,131 kW at 66 revolutions per minute (rpm). The vessel also has three Yanmar/Anqing CSSC 6DK-20e medium-speed, four-stroke auxiliary engines (AEs) of 980 kW rated output at 900 rpm, also IMO Tier II compliant. The ME drives the propeller shaft directly, as shown in Figure S2.

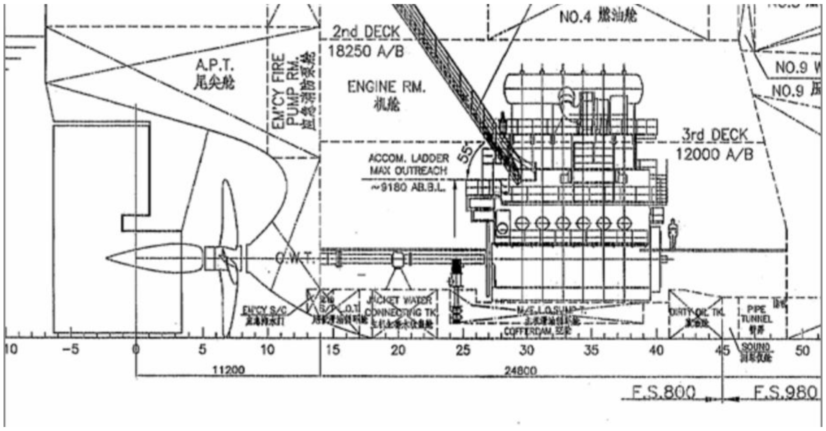

Figure S2: Snippet of the vessel's propulsion system diagram showing that the ME is driving the propeller shaft directly.

The vessel's engines are connected to an open-loop, inline GMT-R-0152 scrubber system, manufactured by Yara Marine Technologies AB, using seawater to remove SO_2. The efficiency of SO_2 removal depends on the flow rates of the exhaust gases and seawater, with no alkali addition. Seawater is pumped from the sea chest to the scrubber tower via the washwater process pumps. Lances with nozzles are placed inside the scrubber tower to spray the exhaust gases with washwater. The washwater inlet valves are remotely controlled, and as the engine load increases, more inlet valves are opened to ensure that enough water is provided for the scrubbing process. Inside the scrubber tower, the washwater is drained through remotely controlled valves, which have to be open all the time during scrubber operation. The demister, consisting of a wire mesh metallic grid, is a component located near the outlet of the scrubber tower. It removes entrained water droplets from the exhaust gas stream after the scrubbing process. The quality of the discharge washwater is continuously monitored using the monitoring water unit. Figure S3 illustrates the main components of the scrubber system.

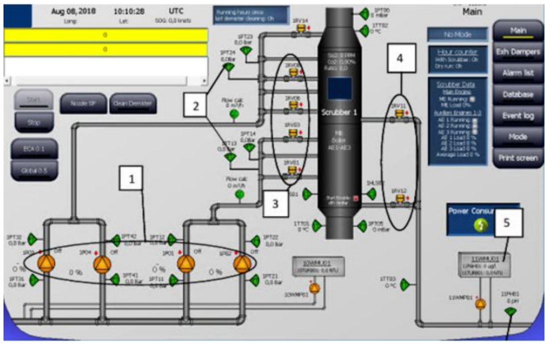

Figure S3: The main components and layout of the scrubber system as displayed on the system's computer interface: 1) washwater process pumps; 2) pressure sensors; 3) remotely controlled inlet valves; 4) remotely controlled drain valves; 5) water monitoring unit.

The vessel is not optimized for a specific fuel, and no adjustments to engine parameters or run-in procedures are required when switching between fuels. The fuel supply system automatically adjusts the treatment of the fuel before injection to ensure that correct injection viscosity is maintained.

The engine back-pressure is slightly affected by the scrubber operation. When the scrubber is in use, the back-pressure increases by a maximum of 5 mbar but remains well within the manufacturer's specified limit of 35 mbar.

There is no evidence to suggest that the scrubber operation impacts the combustion efficiency of the engine.

Note 6 │ Chemical Analyses of Fuels, ME Cylinder Oils & AE Lubricants

Results from the chemical testing of MGO, VLSFO and HFO samples taken during the emission monitoring campaign, including the test methods followed for their analyses, are provided in Tables S2 and S3 below. All fuels were also analyzed before bunkering and these results are shown in Fig. S4-S7. Results from the chemical testing of ME cylinder oils and AE lubricant are provided in Table S4.

Table S2. Chemical analysis of MGO sample collected onboard.

No.	Parameter	Methodology	Unit	MGO
1	Sulfur (S)	ISO 8754	%m/m	0.084
2	Carbon(C)	ASTM D 5291	%m/m	86.89
3	Hydrogen (H)	ASTM D 5291	%m/m	12.99
4	Nitrogen (N)	ASTM D 4629	%m/m	0.031
5	Oxygen (O)	Calculation	%m/m	0.005
6	Water	ASTM D 6304	mg/kg	41
7	Density at 15°C	ISO 12185	kg/m3	855.9
8	Viscosity at 40°C	ISO 3104	cSt	4.999
9	Atmosp. Distillation	ISO 3405		completed
10	Initial Boiling Point	ISO 3405	^{o}C	181.0
11	10% recovery	ISO 3405	^{o}C	251.2
12	50% recovery	ISO 3405	^{o}C	313.4
13	90% recovery	ISO 3405	^{o}C	377.1
14	95% recovery	ISO 3405	^{o}C	391.6
15	Final Point	ISO 3405	^{o}C	396.6
16	Recovery at 250°C	ISO 3405	%vol	9.5
17	Recovery at 350°C	ISO 3405	%vol	74.6
18	Aluminum (Al)	ASTM D 7111	mg/kg	0.3
19	Calcium (Ca)	ASTM D 7111	mg/kg	<0.1
20	Iron (Fe)	ASTM D 7111	mg/kg	<0.1
21	Sodium (Na)	ASTM D 7111	mg/kg	0.1
22	Nickel (Ni)	ASTM D 7111	mg/kg	<0.1
23	Phosphorous (P)	ICP-MS-mod	mg/kg	0.2
24	Silicon (Si)	ASTM D 7111	mg/kg	<0.1
25	Vanadium (V)	ASTM D 7111	mg/kg	<0.1
26	Zinc (Zn)	ASTM D 7111	mg/kg	<0.1

No.	Parameter	Methodology	Unit	MGO
27	Magnesium (Mg)	ASTM D 7111	mg/kg	<0.1
28	Lead (Pb)	ASTM D 7111	mg/kg	0.3
29	Potassium (K)	ASTM D 7111	mg/kg	<0.1
30	Copper (Cu)	ASTM D 7111	mg/kg	0.2
31	Arsenic (As)	ICP-MS-mod	mg/kg	<0.1
32	Barium (Ba)	ASTM D 7111	mg/kg	<0.1
33	Cadmium (Cd)	ICP-MS-mod	mg/kg	<0.1
34	Chromium (Cr)	ASTM D 7111	mg/kg	<0.1
35	Cobalt (Co)	ICP-MS-mod	mg/kg	<0.1
36	Lithium (Li)	ASTM D 7111	mg/kg	<0.1
37	Manganese (Mn)	ASTM D 7111	mg/kg	<0.1
38	Mercury (Hg)	ICP-MS-mod	mg/kg	<0.1
40	Selenium (Se)	ICP-MS-mod	mg/kg	<0.1
41	Titanium (Ti)	ASTM D 7111	mg/kg	<0.1

Table S3. Chemical analyses of VLSFO and HFO samples collected onboard.

No.	Parameter	Methodology	Unit	HFO	VLSFO
1	Sulfur (S)	ISO 8754	%m/m	3.08	0.48
2	Carbon(C)	ASTM D 5291	%m/m	85.38	87.42
3	Hydrogen (H)	ASTM D 5291	%m/m	11.05	11.60
4	Nitrogen (N)	ASTM D 5291	%m/m	0.38	0.31
5	Oxygen (O)	Calculation	%m/m	0.11	0.19
6	Water	ISO 3733	%m/m	0.10	0.05
7	Density at 15°C	ISO 12185	kg/m^3	972.8	946.2
8	Viscosity at 50°C	ISO 3104	cSt	320.0	137.8
9	Aluminum (Al)	IP 501	mg/kg	2	1
10	Calcium (Ca)	IP 501	mg/kg	9	3
11	Iron (Fe)	IP 501	mg/kg	10	5
12	Sodium (Na)	IP 501	mg/kg	28	1
13	Nickel (Ni)	IP 501	mg/kg	26	10
14	Phosphorous (P)	IP 501	mg/kg	<1	<1
15	Silicon (Si)	IP 501	mg/kg	3	2
16	Vanadium (V)	IP 501	mg/kg	140	8
17	Zinc (Zn)	IP 501	mg/kg	1	2
18	Magnesium (Mg)	IP 501mod	mg/kg	1	<1
19	Lead (Pb)	IP 501mod	mg/kg	<1	1
20	Potassium (K)	IP 501mod	mg/kg	1	3
21	Copper (Cu)	IP 501mod	mg/kg	2	<1
22	Arsenic (As)	ICP-MS-mod	mg/kg	<0.1	<0.1
23	Barium (Ba)	ICP-MS-mod	mg/kg	<0.1	<0.1
24	Cadmium (Cd)	ICP-MS-mod	mg/kg	<0.1	<0.1
25	Chromium (Cr)	ICP-MS-mod	mg/kg	<0.1	<0.1
26	Cobalt (Co)	ICP-MS-mod	mg/kg	<0.1	<0.1
27	Lithium (Li)	ICP-MS-mod	mg/kg	<0.1	<0.1
28	Manganese (Mn)	ICP-MS-mod	mg/kg	<0.1	<0.1
29	Mercury (Hg)	ICP-MS-mod	mg/kg	<0.1	<0.1
31	Selenium (Se)	ICP-MS-mod	mg/kg	<0.1	<0.1
32	Titanium (Ti)	ICP-MS-mod	mg/kg	<0.1	<0.1

Fuel Quality Report

HEDWIG OLDENDORFF (9742728)

Zhoushan • 15/04/2023

Sample information has been collated from the vessel's bunker data form and sample bottle label.

**Sample No.**	SH12199	**Grade Ordered**	DMA
**Client**	Oldendorff Carriers	**Sulphur Grade**	0.10% max
**Received by Lab**	23/04/2023	**Quantity Supplied**	180 M.T.
**Terminal**			

Bunker Information	
**Bunker Port**	Zhoushan
**Bunker Date**	15/04/2023
**Sample Date**	15/04/2023
**Fuel Supplier**	Chimbusco
**Barge**	Yi Da 5
**Sample Location**	Ship's bunker line
**Sample Method**	Continuous drip

Sample Seal Information	
**Seal Condition**	Intact
**Analysis Sample**	0230502/0355335
**Supplier Sample**	
**Vessel Sample**	
**Marpol Sample**	

Courier Information	
**DHL PAD Used**	No
**DHL PAD Comments**	
**Courier**	
**Airway Bill No.**	

	Density (kg/m³@15°C)	Viscosity (cSt@40°C)	Sulphur (%mass)
**Specification**	890.0	2.000 - 6.000	0.10
**Supplier BDR**	858.6	3.735	0.039
**Analysed sample**	849.8	3.532	0.034

Parameter	Result	Units	Spec Limit	Test Variance (±)	Method
Viscosity (40°C)	3.532	cSt@40°C	2.000 - 6.000	0.03400	ISO 3104[a]^ (20)
Density	849.8	kg/m³@15°C	890.0 max	0.3	ISO 12185[a]^ (96)
Cetane Index	50.9		40.0 min		ISO 4264 (18)
Sulphur	0.034	% mass	0.10 max	0.010	ISO 8754[a] (03)
Flash Point	>70.0	°C	60.0 min		ISO 2719[a]^ (16)
Acid Number	<0.20	mg KOH/g	0.50 max	0.13	ASTM D664 (18)
Micro Carbon Residue	<0.01	% mass	0.30 max	0.07	ISO 10370[a] (14)
Cloud Point	+2	°C			ISO 3015 (19)
Cold Filter Plugging Point	-2	°C			IP 309
Pour Point	-6	°C	0 max	4	ISO 3016 (19)
Appearance	Clear/Bright				VISUAL
Water	<0.05	% vol			ISO 3733 (99)
Ash	0.001	% mass	0.010 max	0.003	ISO 6245 (01)
Lubricity Wear Scar Diameter	438	μm	520 max	60	ISO 12156-1:A (18)
Net Specific Energy	42.76	MJ/kg			ISO 8217:A
Fatty Acid Methyl Ester	0.1	%	0.5 max		IP 579 (14)

Sample results are compared with the specification for DMA ISO 8217:2010 for the parameters reported.

Figure S4. Chemical analysis of MGO before bunkering.

Analysis Report: NB23-06915.001

Report Date: 17/05/2023

OLDENDORFF CARRIERS GMBH & CO. KG.
WILLY-BRANDT-ALLEE 6
LUBECK
GERMANY
D-23554

The results shown in this test report specifically refer to the sample(s) tested as received unless otherwise stated. All tests have been performed using the latest revision of the methods indicated, unless specifically marked otherwise on the report. Precision parameters apply in the determination of the below results. Users of analytical results, when establishing conformance with commercial or regulatory requirements should note the full provisions of ASTM D3244, IP 367 and ISO 4259 in that context, the default confidence level of petroleum testing having been set at the 95% confidence level. Your attention is specifically drawn to Sections 7.3.6., 7.3.7 and 7.3.8 of ASTM D3244. With respect to the UOP methods listed in the report below the user is referred to the method and the statement within it specifying that the precision statements were determined using UOP Method 999. This Test Report is issued under the Company's General Conditions of Service (copy available upon request or on the company website at www.sgs.com). Attention is drawn to the limitations of liability, indemnification and jurisdictional issues defined therein. This report shall not be reproduced except in full, without the written approval of the laboratory.

JOB ORDER NO. :	COBONB2300889-01FO	BOSS ORDER NO.:	--
CLIENT ID :	N/A	VESSEL :	HEDWIG OLDENDORFF
LOCATION :	Beilun,Ningbo	PRODUCT DESCRIPTION :	Fuel Oil - LSFO
SAMPLE SOURCE :	Ship Manifold	SOURCE ID :	N/A
SAMPLE TYPE :	During Transfer	SAMPLE BY :	SGS
SAMPLED :	15/05/2023	RECEIVED :	16/05/2023
ANALYSED :	16/05/2023 - 17/05/2023	COMPLETED :	17/05/2023
CONTAINER:	1×1L Plastic Bottle	SAMPLE STATE:	Black Liquid
SEAL NO.:	3218311	SUPPLY SHIP:	YI DING 7

REPORT COMMENT :
1.The fuel shall be free from ULO.A fuel shall be considered to contain ULO when either one of the following conditions is met:
--calcium>30 and zinc>15;or
--calcium>30 and phosphorus>15.
2.The test report shall only be used for clients' scientific research, teaching, internal quality control, product research and development, etc... and just for internal reference.
Fuel oil suppliers of the ship provide 15°C density is: 0.9464g/cm3.

PROPERTY	METHOD	RESULT	UNITS	MIN	MAX
Kinematic Viscosity at 50°C	ISO 3104:2020	189.1	mm²/s	--	380.0
Density at 15°C	ISO 3675:1998	946.2	kg/m³	--	991.0
Calculated Carbon Aromaticity Index	ISO 8217:2017(Annex C)	815	---	--	870
Sulfur	ISO 8754:2003	0.45	% (m/m)	--	0.50
Flash Point	ISO 2719:2016+Amd1 -2021(Method B)	>100	°C	60.0	--
Hydrogen Sulfide content - (Procedure A)	IP 570/15(2021)	<0.5	mg/kg	--	2.0
Acid Number (Inflection end-point)	ASTM D664-18e2(Method A)	0.24	mg KOH/g	--	2.5
Potential Total Sediment - TSP	ISO 10307-2:2009/Cor.1:2010(Procedure A)	0.02	% (m/m)	--	0.10
Carbon Residue - Micro Method	ISO 10370:2014	6.9	% (m/m)	--	18.0
Upper Pour Point	ISO 3016:2019	3	°C	--	30
Water Content	ISO 3733:1999	0.10	% (V/m)	--	0.50
Ash	ISO 6245:2001	0.009	% (m/m)	--	0.100
Elements	IP 501/05(2019)				
Aluminium		5	mg/kg	--	--
Silicon		<10	mg/kg	--	--
Aluminium + Silicon		<15	mg/kg	--	60
Vanadium		8	mg/kg	--	350
Calcium		7	mg/kg	--	--
Phosphorus		<1	mg/kg	--	--
Zinc		<1	mg/kg	--	--
Sodium		5	mg/kg	--	100

-------------------------------- End of Analytical Results --------------------------------

Figure S5. Chemical analysis of VLSFO before bunkering.

Fuel Quality Report

HEDWIG OLDENDORFF (9742728)

Zhoushan • 15/04/2023

Overview

The sample results indicate that all parameters are within the specification limit.

Sample information has been collated from the vessel's bunker data form and sample bottle label.

**Sample No.**	SH12196	**Grade Ordered**	RMG380
**Client**	Oldendorff Carriers	**Sulphur Grade**	3.50% max
**Received by Lab**	23/04/2023	**Quantity Supplied**	900 M.T.
**Terminal**			

Bunker Information	
**Bunker Port**	Zhoushan
**Bunker Date**	15/04/2023
**Sample Date**	15/04/2023
**Fuel Supplier**	Chimbusco
**Barge**	Yi Da 5
**Sample Location**	Ship's bunker line
**Sample Method**	Continuous drip

Sample Seal Information	
**Seal Condition**	Intact
**Analysis Sample**	0355331/0168007
**Supplier Sample**	0355333/0168009
**Vessel Sample**	0355332/0168008
**Marpol Sample**	0355334/0168010

Courier Information	
**DHL PAD Used**	No
**DHL PAD Comments**	
**Courier**	
**Airway Bill No.**	

	Density (kg/m³@15°C)	Viscosity (cSt@50°C)	Sulphur (%mass)
**Specification**	991.0	380.0	3.50
**Supplier BDR**	977.1	355.6	2.62
**Analysed sample**	976.6	368.4	2.70

Parameter	Result	Units	Spec Limit	Test Variance (±)	Method
Viscosity (50°C)	368.4	cSt@50°C	380.0 max	18.97	ISO 3104[a]^ (20)
Density	976.6	kg/m³@15°C	991.0 max	0.9	ISO 12185[a]^ (96)
CCAI	838	Index #	870 max		ISO 8217:B
Sulphur	2.70	% mass	3.50 max	0.17	ISO 8754[a] (03)
Flash Point	>70.0	°C	60.0 min		ISO 2719[a]^ (16)
Acid Number	0.22	mg KOH/g	2.50 max	0.50	ASTM D664 (18)
Total Sediment Existent	0.01	% mass	0.10 max	0.03	ISO 10307-1 (09)
Total Sediment	0.01	% mass	0.10 max	0.05	ISO 10307-2 (09)
Micro Carbon Residue	11.97	% mass	18.00 max	0.99	ISO 10370[a] (14)
Pour Point	0	°C	30 max	4	ISO 3016 (19)
Water	0.25	% vol	0.50 max	0.12	ISO 3733 (99)
Ash	0.039	% mass	0.100 max	0.014	ISO 6245 (01)
Vanadium	123	mg/kg	350 max	33	IP 501 (05)
Sodium	8	mg/kg	100 max	8	IP 501 (05)
Aluminium plus Silicon	4	mg/kg	60 max	12	IP 501 (05)

Figure S6. Chemical analysis of HFO before bunkering.

Parameter	Result	Units	Spec Limit	Test Variance (±)	Method
Net Specific Energy	40.41	MJ/kg			ISO 8217:A
Calcium	2	mg/kg			IP 501 (05)
Zinc	<1	mg/kg			IP 501 (05)
Phosphorus	<1	mg/kg			IP 501 (05)
Aluminium	3	mg/kg			IP 501 (05)
Silicon	1	mg/kg			IP 501 (05)
Iron	17	mg/kg			IP 501 (05)
Nickel	32	mg/kg			IP 501 (05)
Compatibility	1	Spot #			ASTM D4740 (20)
Injection Temp @ 10 cSt	144	°C			
Injection Temp @ 12 cSt	137	°C			
Injection Temp @ 15 cSt	128	°C			
Injection Temp @ 17 cSt	123	°C			
Injection Temp @ 20 cSt	118	°C			
Injection Temp @ 22 cSt	115	°C			
Minimum Pumping Temp @1000 cSt	34	°C			
Headspace GCMS Screening	Green				
Asphaltene	6.7	% mass			IP 143 (16)
Reserve Stability Number	10.9				ASTM D7061 (19)

Figure S7. Chemical analysis of HFO before bunkering – cont'd.

Table S4. Chemical analyses of ME cylinder oils and AE lubricant samples collected onboard.

No.	Parameter	Methodology	Unit	ME Cylinder oil for MGO	ME Cylinder oil for VLSFO &HFO	Lubricant AE1	Clean AE lubricant	Lubricant AE2
1	Sulfur (S)	ISO 8754	%m/m	0.34	0.46	0.24	0.24	0.44
2	Carbon(C)	ASTM D 5291	%m/m	82.3	79.07	83.7	85.06	84.3
3	Hydrogen (H)	ASTM D 5291	%m/m	12.6	12	12.7	13.11	12.99
4	Nitrogen (N)	ASTM D 5291	%m/m	0.21	0.2	0.177	0.296	0.293
5	Oxygen (O)	Calculation	%m/m	4.55	8.27	3.183	1.294	1.977
6	Water	ASTM D 6304	mg/kg	0.19	0.04	0.08	0.08	0.06
7	Aluminum (Al)	ASTM D 5185	mg/kg	4	7	3	2	2
8	Calcium (Ca)	ASTM D 5185	mg/kg	19714	36882	10894	12129	11199
9	Iron (Fe)	ASTM D 5185	mg/kg	3	6	14	2	16
10	Sodium (Na)	ASTM D 5185	mg/kg	2	4	19	2	20
11	Nickel (Ni)	ASTM D 5185	mg/kg	<1	<1	21	<1	22
12	Phosphorous (P)	ASTM D 5185	mg/kg	3	2	222	236	222
13	Silicon (Si)	ASTM D 5185	mg/kg	38	49	6	11	6
14	Vanadium (V)	ASTM D 5185	mg/kg	<1	1	77	<1	80
15	Zinc (Zn)	ASTM D 5185	mg/kg	18	23	232	253	235
16	Magnesium (Mg)	ASTM D 5185	mg/kg	52	98	29	32	30
17	Lead (Pb)	ASTM D 5185	mg/kg	<1	<1	<1	<1	<1
18	Potassium (K)	ASTM D 5185	mg/kg	<2	<2	<2	<2	<2
19	Copper (Cu)	ASTM D 5185	mg/kg	<1	<1	3	<1	<1
20	Arsenic (As)	ICP-MS-mod	mg/kg	<0.2	<0.2	<0.2	<0.2	<0.2
21	Barium (Ba)	ASTM D 5185	mg/kg	<1	<1	<1	<1	<1
22	Cadmium (Cd)	ICP-MS-mod	mg/kg	<0.2	<0.2	<0.2	<0.2	<0.2
23	Chromium (Cr)	ASTM D 5185	mg/kg	<1	<1	<1	<1	<1
24	Cobalt (Co)	ICP-MS-mod	mg/kg	<0.2	<0.2	<0.2	<0.2	<0.2

No.	Parameter	Methodology	Unit	ME Cylinder oil for MGO	ME Cylinder oil for VLSFO &HFO	Lubricant AE1	Clean AE lubricant	Lubricant AE2
25	Lithium (Li)	ICP-MS-mod	mg/kg	<0.2	<0.2	<0.2	<0.2	<0.2
26	Manganese (Mn)	ASTM D 5185	mg/kg	2	5	2	2	2
27	Mercury (Hg)	ICP-MS-mod	mg/kg	<0.2	<0.2	<0.2	<0.2	<0.2
29	Selenium (Se)	ICP-MS-mod	mg/kg	<0.2	<0.2	<0.2	<0.2	<0.2
30	Titanium (Ti)	ASTM D 5185	mg/kg	<1	<1	<1	<1	<1

Note 7 | Engine & Scrubber Operating Modes

Four engine operating modes were specified: idle, and at 25%, 50%, and 80% of the ME's load (% MCR). Weighting factors for each mode were determined based on hourly engine output data (% MCR) for Hedwig Oldendorff collected from January 2022 to October 2023. During this period, the ME was in idle for 37% of the time, operated at ≤35% load for about 12% of the time, at 35-60% load for 43%, and at >60% load for 8% of the time, reaching a maximum of about 88% MCR. The engine operating modes, their respective weighting factors, and recorded engine and scrubber conditions are presented in Table S5.

The scrubber can operate in two modes: the global 0.5% S cap and the ECA 0.1% S cap. For all four engine operating conditions, the scrubber was set to the 0.5% S cap mode, as this is more common in practice. This mode also results in potentially higher washwater concentrations due to lower water flow rates pumped through the system compared to the 0.1% S mode, ensuring our findings represent typical and worst-case operating conditions.

Table S5. Engine operating modes and relevant engine conditions per tested fuel.

	Mode 1:<br>ME idle	Mode 2:<br>ME ~25%	Mode 3:<br>ME ~50%	Mode 4<br>ME ~80%
Weighting factor	37%	12%	43%	8%
Engine conditions while burning MGO				
Engine load (%)	ME: 0%<br>AE1: 51%<br>AE2: 47%	ME: 25%<br>AE1: 37%<br>AE2: 36%	ME: 54%<br>AE1: 33%<br>AE2: 32%	ME: 80%<br>AE1: 33%<br>AE2: 32%
ME speed (rpm)*	-	42	54	61
Generated power (kW)	957	4,502	8,805	12,742
Consumed fuel (kg h^{-1})	230	790	1,400	2,040
Specific fuel consumption (g kW^{-1} h^{-1})	240	175	159	160
Engine conditions while burning VLSFO				
Engine load (%)	ME: 0%<br>AE1: 51%<br>AE2: 49%	ME: 25%<br>AE1: 37%<br>AE2: 37%	ME: 48%<br>AE1: 35%<br>AE2: 35%	ME: 78%<br>AE1: 39%<br>AE2: 38%
ME speed (rpm)*	-	41	53	60
Generated power (kW)	982	4,512	7,930	12,563
Consumed fuel (kg h^{-1})	230	850	1,390	2,040
Specific fuel consumption (g kW^{-1} h^{-1})	234	188	175	162
Engine conditions while burning HFO				
Engine load (%)	ME: 0%<br>AE1: 60%<br>AE2: 59%	ME: 25%<br>AE1: 39%<br>AE2: 38%	ME: 50%<br>AE1: 45%<br>AE2: 45%	ME: 80%<br>AE1: 49%<br>AE2: 49%
ME speed (rpm)*	-	43	53	61
Generated power (kW)	1,169	4,539	8,373	13,067
Consumed fuel (kg h^{-1})	270	810	1,460	2,200
Specific fuel consumption (g kW^{-1} h^{-1})	231	178	174	168
Scrubber load	7%	30%	55%	85%

*AEs were running continuously at 900 rpm regardless of the load.

Note 8 │ Gas & PM Measurements

All gas-related measurements were performed using the P2000 Continuous Emission Monitoring Systems (CEMS) (Protea Ltd, United Kingdom). These systems were stack-mounted at the particular sampling points upstream and downstream of the scrubber (Figures S8 and S9), minimizing potential uncertainties and have been designed for such environments. They are designed to provide in-situ measurements for such environments, withstanding sample temperatures up to 350°C (~100 oC higher than the exhaust gas temperature upstream of the scrubber)[15].

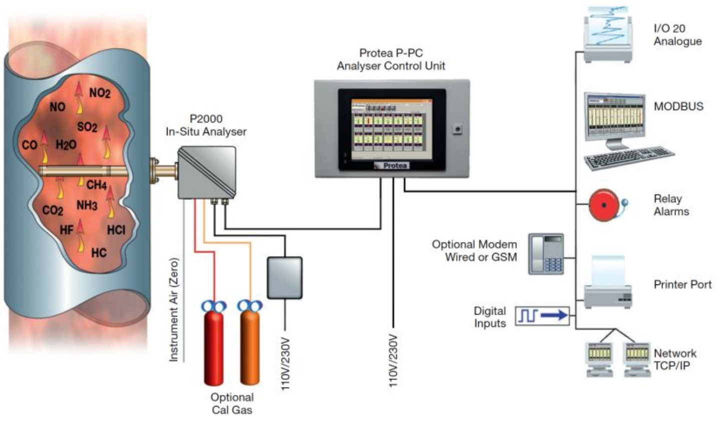

Figure S8. Basic components and schematic of the P2000 (CEMS)[15].

For $PM_{2.5}$ measurements, a perforated sampling probe was installed near the gaseous emissions measurement instruments, but sufficiently distant to not cause any interference. A heated sampling line was used to transport collected exhaust gas to the eDiluter™ Pro for dilution and conditioning. We had adjusted the temperature of this heated line to 260 ^{o}C upstream and 200 ^{o}C downstream of the scrubber (same temperature as the first dilution stage of the eDiluter™ Pro). The measured water vapor (H_2O) concentrations were ~ 4% vol.

upstream of the scrubber and ~ 3% vol. downstream of the scrubber. Based on these values, we calculated the dew points in the sampling lines to be approximately 29 °C upstream and 25 °C downstream of the scrubber. These dew points are well below the adjusted temperatures of the heated sampling line, ensuring that no condensation occurred during sampling. From the dilutor, the exhaust gas was connected to the TSI DustTrak using a short (less than 1 m) black carbon-impregnated silicone tubing to minimize PM losses.

The dilution instrument used is not designed to heat the sample, but rather to maintain its temperature and prevent it from decreasing. This prevents temperature drops that could lead to water vapor condensation, which is critical downstream of the scrubber. Moreover, due to the very short residence time and the use of the lowest possible dilution ratio (5) used in this stage, the actual sample temperature is expected to be well below 200 °C. Given that the volatility threshold of HFO- and lubricant-derived semi-volatile particles and sulfates typically starts at 200 °C or higher, significant evaporation under these conditions is unlikely.

A) B)

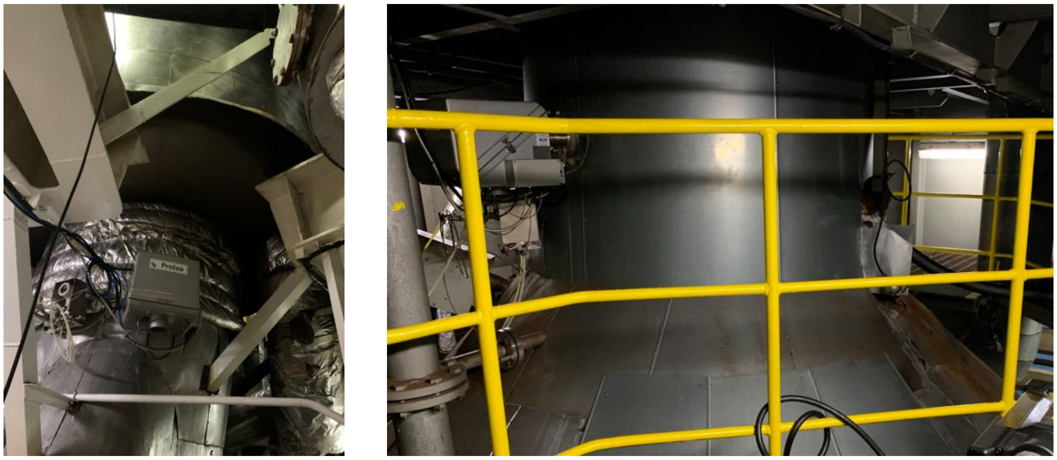

Figure S9. The P2000 CEMS analyzers A) upstream and B) downstream of the scrubber.

The Dekati dilutor and the TSI DustTrak, used for $PM_{2.5}$ mass measurements, were located outside the stack, in close proximity to the areas surrounding the inlet and outlet of the scrubber tower for upstream and downstream measurements, respectively (Figure S10). The temperature at these locations were ~ 35-45 ^{o}C. Both instruments can safely operate up to 50 ^{o}C according to their manuals. This was further confirmed though discussions with the respective manufacturers.

A) B)

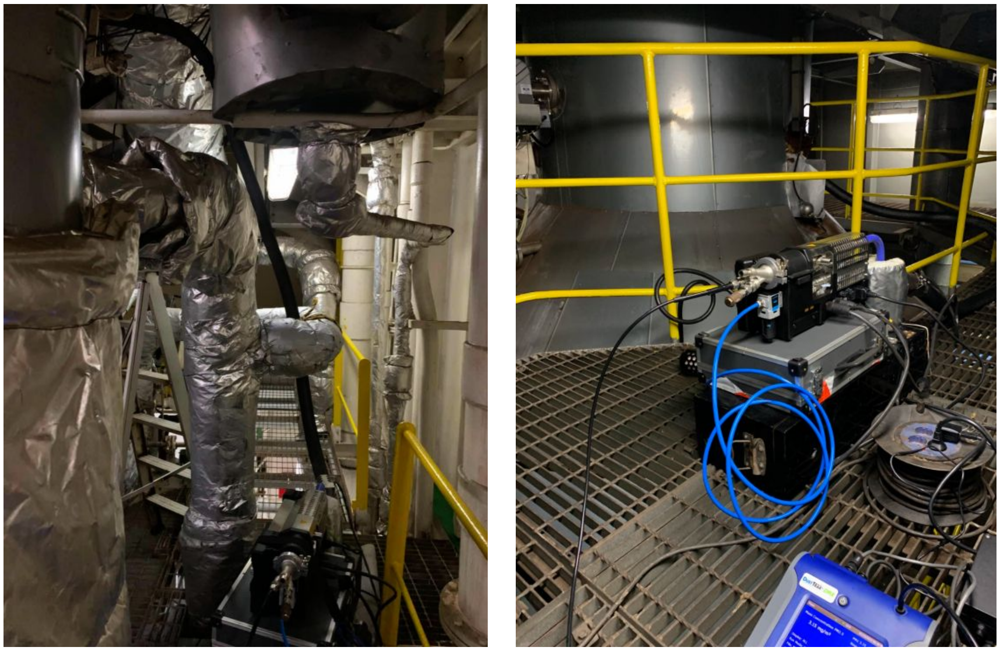

Figure S10. The Dekati dilutor and the TSI DustTrak instruments during measurements A) upstream and B) downstream of the scrubber.

The TSI DustTrak was chosen as the most practical tool to provide valuable real-time data for comparing $PM_{2.5}$ mass emissions across fuels under real-world conditions, given the challenges of performing gravimetric measurements onboard. However, for future studies, the use of weighing filters in a laboratory gravimetry chamber prior/after the campaign to

derive accurate $PM_{2.5}$ mass values and correction factors for the TSI DustTrak specific for each fuel and load would be recommended. Simultaneous measurements of size distributions (including sub-100 nm), using *e.g.,* SMPS/ELPI/DMS500, would significantly improve PM characterization. Although the TSI DustTrak has a built-in filter holder for gravimetric correction, an external sampler may be needed to collect sufficient aerosol mass for accurate gravimetry from short-duration measurements.

Measurements were performed on a wet basis, and the relevant emission factors presented refer to wet concentrations, following the methodology described in ISO 8178:4, section 9.1.4.2.1[16]. The measurements downstream of the scrubber were conducted after the demister, a component designed to remove water droplets from the exhaust gas stream post-scrubbing. As a result, the measured water vapor content downstream of the scrubber (~ 3% vol.) is close to or slightly lower than the levels measured upstream of the scrubber (~ 4% vol.).

Additionally, the scrubbing process leads to a significant temperature drop in the exhaust gas (from ~ 220°C upstream to ~ 30°C downstream). This cooling effect reduces the volume of the exhaust gas downstream of the scrubber. The reduced gas volume has been accounted for in the calculations of the emission factors, ensuring that the downstream emissions are accurately represented.

Note 9 | Seawater & Washwater Sample Collection & Preservation

Samples of seawater inlet and washwater overboard discharge were collected from onboard monitoring stations using existing manual valves (Fig. S11, S12). No treatment, dilution, or pH adjustment of seawater or washwater occurred before entering the scrubber system or before discharge into the sea, respectively. Seawater inlet samples preceded washwater samples. The residence time of water inside the scrubber system is very short; seawater is practically used directly upon entering the system and transformed into washwater within minutes as the system operates continuously. Thus, there was no need to wait between sampling points.

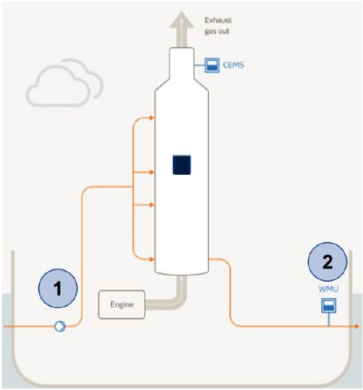

Figure S11. The two scrubber sampling points: 1) seawater inlet; 2) ww overboard discharge.

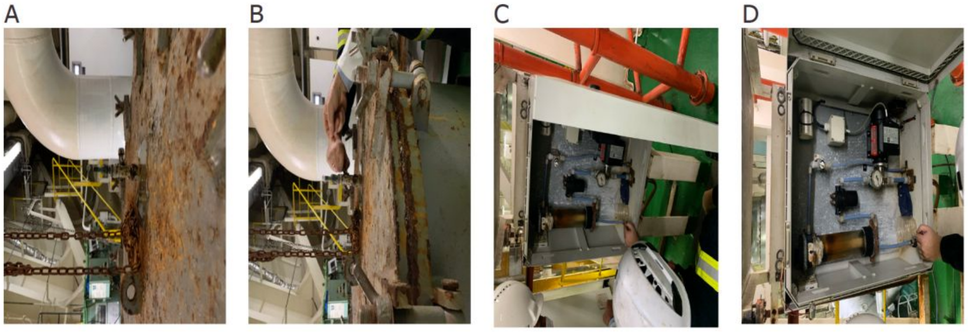

Figure S12. A & B) The 1st sampling point, *i.e.* seawater inlet; C & D) The 2nd sampling point, *i.e.* washwater overboard discharge.

The NAIAs seawater/ww sampling kits[17] were used, provided inside blue large cooler boxers. Each kit/cooler box contained 2 zip-lock bags, 1 bag per sampling point (Fig. S13). Each bag contained:

- 1 bottle for Nitrates-Nitrites;
- 1 bottle for Total Metals;
- 1 bottle for Dissolved Metals;
- 1 amber glass bottle with Teflon lid for PAHs;
- Blue ice packs (to be taken out and kept in the freezer);
- Samples identification form completed for each pair of sampling points.

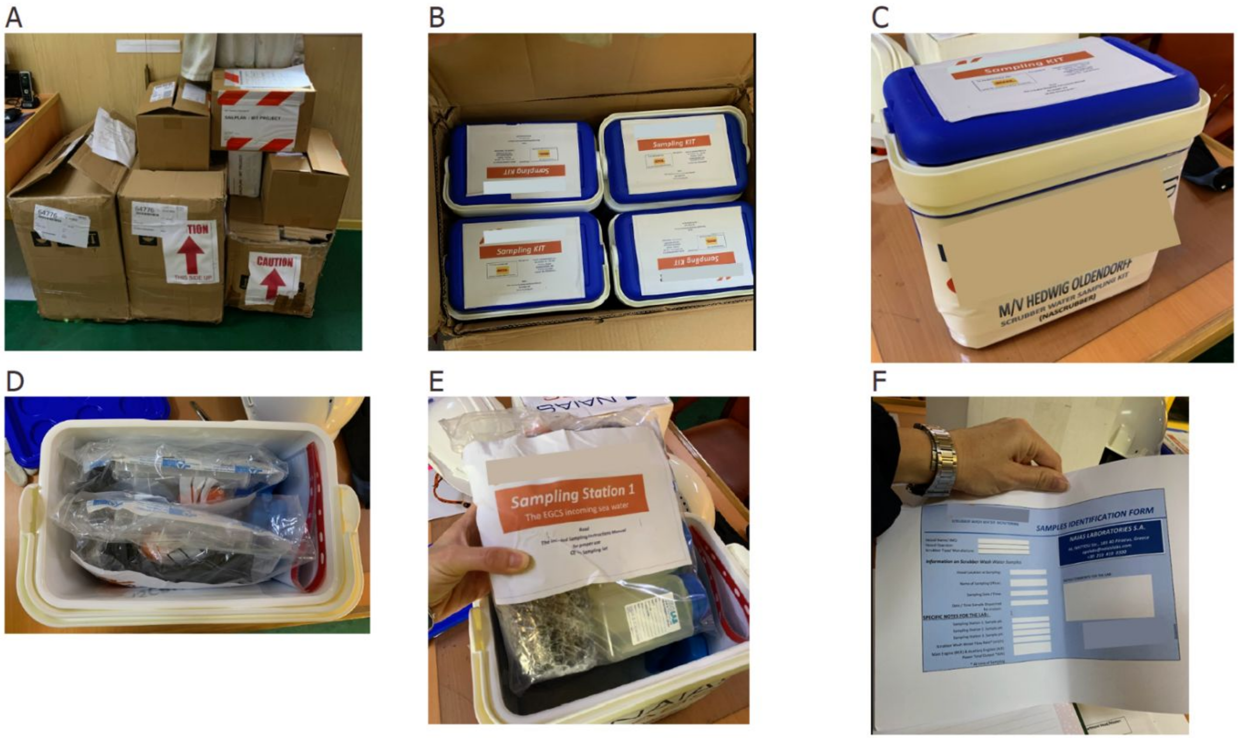

Figure S13. The sampling kits used.

Apart from the NAIAS sampling kits, the following field test equipment was used:

- *Handheld pH meter (Fig. S14A)*: The pH meter has a resolution of 0.1 pH units and temperature compensation. It has been calibrated and it meets the requirements defined in BS 2586 and BS EN ISO 60746-2:2003.

- *Total Residual Oxidants (TRO) monitoring equipment (Fig. S14B)*: TRO is most commonly used as an umbrella term referring to disinfection by-products including hypochlorite, chlorite, chlorate and chlorine dioxide. It may also include chlorine if chlorinated water is being treated with chlorine dioxide ClO2. While TRO is not identified as a required analyte in the US EPA VGP, it is needed to determine if preservation is needed for PAHs samples.

- *Portable Turbidity meter (Fig. S14C)*: Meets requirements defined in ISO 7027:1999 or USEPA 180.1.

- Amber glass bottles for storing samples for oil-in-water measurements.

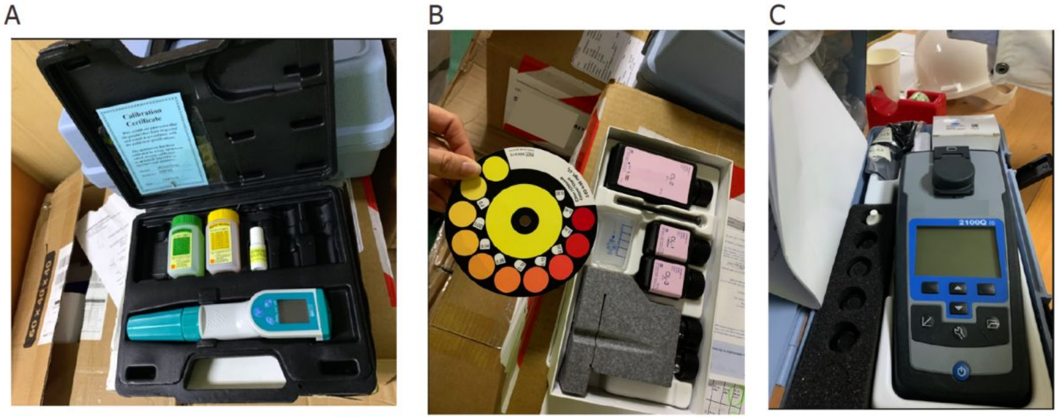

Figure S14. A) pH meter; B) TRO monitoring equipment; C) Turbidity meter.

All sample bottles supplied were cleaned and ready for immediate sampling, while all equipment used for field measurements were calibrated following applicable calibration procedures specified by the instrument manufacturers.

Seawater and washwater samples were collected during the four engine operating modes described in the manuscript, while burning HFO and running the scrubber. Samples were

collected after running the ME and the scrubber system for at least 30 mins in each mode to ensure that they have reached steady state. Sampling points were thoroughly flushed before each sampling event, with at least 10 L of water to remove impurities. 5 L samples from both inlet and outlet points were collected in single-use, disposable plastic buckets and immediately transferred to the owner's cabin for preparation and storage.

The owner's cabin was equipped with a dedicated sample preparation area. The cabin's table was covered with a waterproof plastic tablecloth to avoid any liquid spillage. Relevant Personal Protective Equipment (PPE), including protective gloves, lab coat and eye protection, was used as acid preservatives and chemicals were used for sample preparation.

Within 15 minutes of sample collection, pH, turbidity, and the potential presence of Total Residual Oxidants (TRO) were measured using the ISOLAB portable pH, mV, and temperature meter (Isolab Laborgeräte GmbH, Eschau, Germany)[18], the HACH 2100Q*is* turbidity meter (HACH, Loveland, Colorado, USA)[19], and the Macherey-Nagel VISOCOLOR HE colorimetric test kit for chlorine at low concentrations (Macherey-Nagel GmbH & Co. KG, Düren, Germany)[20] respectively. TRO is used as an umbrella term referring to disinfection by-products like hypochlorite, chlorite, chlorate, and chlorine dioxide, and may also include chlorine if chlorinated water is treated with chlorine dioxide. TRO is necessary to ascertain the need for preservation in PAHs samples. As no TRO was found in any sample, no relevant preservation was considered necessary. Samples for PAHs monitoring were transferred into amber glass bottles with Teflon lids.

Samples for nitrates-nitrites monitoring were transferred into designated 250 mL plastic bottles and acidified with 4 mL of sulfuric acid (H_2SO_4). Those intended for total metals

monitoring were also transferred into designated 250 mL plastic bottles but acidified with approximately 3 mL of nitric acid (HNO_3) to achieve a pH of ≤ 2. For dissolved metals monitoring, samples underwent filtration using disposable vacuum filtration bottles with a pore size of 0.45 μm. The filtered samples were then transferred into designated 250 mL plastic bottles and acidified with approximately 3 mL of HNO_3 to maintain a pH of ≤ 2. For both sample points, samples were transferred to amber glass bottles for oil-in-water monitoring. All sealed bottles were refrigerated until being dispatched overnight to the lab for analysis. Portable cooler boxes equipped with ice packs were used for shipping (2 ice packs in each cooler). Glass bottles were securely enclosed in bubble wrap, while plastic bottles were sealed inside plastic zipper storage bags. All bottles had waterproof labels, and the Samples Identification forms corresponding to each sample and cooler box were thoroughly completed and added in relevant boxes.

All reagents used were of analytical grade and obtained from Sigma-Aldrich (Sigma-Aldrich, Burlington, Massachusetts, USA). They were used as received without any additional purification.

All seawater and washwater samples were collected between May 22-24 and promptly dispatched to the lab upon arrival at the port of Hong Kong on May 25, using an express delivery service. They were delivered to the lab by the morning of May 28. The PAH analyses started right away and were completed within a maximum of 7 days from sample collection for all samples.

Note 10 | Seawater & Washwater Sample Analyses

All analyses were undertaken by the ISO 17025-accredited Naias Labs S.A[21]. For TDS and TSS measurements, a well-mixed sample was first filtered through a standard glass-fiber filter. For TDS, the filtrate was transferred to a pre-weighed dish, evaporated to dryness, and then dried to a constant weight in an oven at 180 °C (Binder, Germany). The increase in weight of the dish compared to its empty pre-weighed state represented the TDS. For TSS, the filter and the residue retained on it were dried to a constant weight in an oven at 103–105 °C (Binder, Germany). The increase in the weight of the filter represented the TSS.

The samples underwent photometric analysis (Gallery Plus Discrete Analyzer, Thermo Fisher Scientific, Waltham, Massachusetts, USA) to measure N, P and S ionic species (NO^{3-}, NO^{2-}, NH^{4+}, PO^{43-}, SO_4^{2-}, SO^{2-}), following ISO 15923-1:2013. Relevant calibration standards were utilized (HACH, Loveland, Colorado, USA). For Kjeldahl N measurements, a digestion system and a distillation system were employed (Velp DKL 12, Velp UDK 139, Velp Scientifica, Lombardy, Italy), as described in ISO 5663:1984.

The PAHs concentrations were determined via Gas Chromatography Mass Spectrometry (GC-MS) (TRACE GC-MS/MS system, Thermo Fisher Scientific, Waltham, Massachusetts, USA) adhering to ISO 28540:2011 and EN 16691:2015, using relevant calibration and internal standards (Supelco, USA). GC-MS was also used to measure the 1-4 dichlorobenzene and BTEX concentrations (DSQ II and TRACE GC-MS/MS system, Thermo Fisher Scientific, Waltham, Massachusetts, USA). The GC-MS was equipped with an automatic sampler and a Solid-Phase Dynamic Extraction (SPDE) extraction cooler (Combi PAL, CTC Analytics AG, Zwingen, Switzerland) according to ISO 17943:2016 and

ISO 20595:2018, using pertinent calibration and internal standards (Thermo Fisher Scientific, Waltham, Massachusetts, USA).

GC - Flame ionization detector (FID) analysis was performed to measure hydrocarbons and mineral oil concentrations (GC-2010 PLUS system with injector PTV and automatic sampler AOC 20i FID, SHIMADZU, Kyoto, Japan), using relevant calibration and internal standards (Supelco, USA), according to ISO 17943:2016 and ISO 20595:2018.

Inductively Coupled Plasma Mass Spectrometry (ICP-MS) was used to measure metal concentrations (8900 Triple Quadrupole ICP-MS system, Agilent, Santa Clara, California, USA), following ISO 17294-1:2004 and ISO 17294-2:2016. Relevant calibration and internal standards were used (Agilent, Santa Clara, California, USA).

Table S6. Chemical parameters analyzed in seawater and washwater samples.

No.	Parameter	Unit
1	pH	pH units
2	Turbidity	FNU
3	Electrical Conductivity	μm/cm
4	TDS	mg/L
5	TSS	mg/L
6	Nitrates (NO^{3-})	mg/L
7	Nitrites (NO^{2-})	mg/L
8	Ammonium (NH_4^+)	mg/L
9	Kjeldahl N	mg/L
10	Total N	mg/L
11	Phosphates (PO_4^{3-})	mg/L
12	Total P	mg/L
13	Sulfates (SO_4^{2-})	mg/L
14	Sulfites (SO_3^{2-})	mg/L
15	Oil in water	μg/L
16	Hydrocarbon oil index (C10-C40)	μg/L
17	1,4-Dichlorobenzene	μg/L
18	Benzene	μg/L

No.	Parameter	Unit
19	Benzo(a)pyrene	µg/L
20	Anthracene	µg/L
21	Benzo(a)anthracene	µg/L
22	Chrysene	µg/L
23	Fluoranthene	µg/L
24	Phenanthrene	µg/L
25	Pyrene	µg/L
26	Fluorene	µg/L
27	Dibenzo(a,h)anthracene	µg/L
28	Benzo(b)fluoranthene	µg/L
29	Benzo(k)fluoranthene	µg/L
30	Benzo(g,h,i)perylene	µg/L
31	Indeno(1,2,3-c,d)pyrene	µg/L
32	Naphthalene	µg/L
33	Acenaphthylene	µg/L
34	Acenaphthene	µg/L
35	Toluene	µg/L
36	Ethylbenzene	µg/L
37	o-Xylene	µg/L
38	m, p-Xylene (sum)	µg/L
39	Aluminum (Al)	µg/L
40	Arsenic (As)	µg/L
41	Barium (Ba)	µg/L
42	Cadmium (Cd)	µg/L
43	Calcium (Ca)	mg/L
44	Chromium (Cr)	µg/L
45	Cobalt (Co)	µg/L
46	Copper (Cu)	µg/L
47	Iron (Fe)	µg/L
48	Lead (Pb)	µg/L
49	Lithium (Li)	µg/L
50	Magnesium (Mg)	mg/L
51	Manganese (Mn)	µg/L
52	Mercury (Hg)	µg/L
53	Molybdenum (Mo)	µg/L
54	Nickel (Ni)	µg/L
55	Potassium (K)	mg/L
56	Selenium (Se)	µg/L

No.	Parameter	Unit
57	Silicon (Si)	mg/L
58	Sodium (Na)	mg/L
59	Thallium (Tl)	μg/L
60	Vanadium (V)	μg/L
61	Zinc (Zn)	μg/L

Measurements of TDS, TSS, N, P, and S ionic species, Kjeldahl N, PAHs, 1,4-dichlorobenzene, BTEX, hydrocarbons, and oil concentrations were conducted in duplicates, while metal concentrations were measured in triplicates. Mean values are reported for all measurements.

Note 11 │ Calculation of Emission Factors

Emissions were measured instantaneously at minute intervals, expressed in parts per million for CO, NO_x, and SO_2, percentage of exhaust gas volume (%v/v) for CO_2, and mass concentrations per exhaust gas volume (mg/m^3) for $PM_{2.5}$. The instantaneous emissions for each parameter were converted to standard conditions and expressed to grams of emissions per kilowatt hour of generated power (g kW^{-1} h^{-1}) to facilitate meaningful comparisons. The gas concentrations were measured on a wet basis and the relevant emission factors presented refer to wet concentrations following the methodology described in ISO 8178:4, section 9.1.4.2.1[16]. This conversion relied on the exhaust gas flow rates (actual and normal), which was determined using specific fuel consumption and emitted CO_2, following the carbon balance method outlined in ISO 8178 recommendations[16]. This methodology is commonly adopted in pertinent literature as well[11,22–25]. For each engine mode, the actual and normal exhaust gas flow rates (Exh_{flow} in m3 h^{-1}) were calculated based on the produced CO_2,

assuming complete conversion of all carbon in the fuels into CO_2 during combustion, as described by Equation (1).

$$Exh_{flow} = \frac{Fuel\_cons. * Fuel\_C\_content * ({}^{MM_{CO_2}}/_{MM_C})}{D_{CO2} * (C_{CO2,\,exh} - C_{CO2,air})} \quad (1)$$

where *Fuel_cons.* is the consumed fuel (kg/h); *Fuel_C_content* is the carbon content of the fuel (% m/m); MM_{CO2} is the molar mass of CO_2 (g/mol); MM_C is the molar mass of carbon (g/mol); D_{CO2} is the density of CO2 (kg/m^3); $C_{CO2,exh}$ is the concentration of CO_2 in the exhaust gas (% v/v); $C_{CO2,air}$ is the concentration of CO_2 in the atmospheric air (% v/v).

The EFs of the monitored gases and $PM_{2.5}$ were subsequently calculated in mass per engine work (g kWh^{-1}) and normalized to standard conditions (temperature 273.15 ^oK and pressure 101.325 kPa) following Equation (2) and Equation (3) respectively.

$$EF_k = ER_k * \frac{P * Exh_{flow} * MM_k}{R * T * Engine\_output} \quad (2)$$

$$EF_{PM_{2.5}} = \frac{Exh_{flow} * PM_{2.5} * DR}{Engine\_output} \quad (3)$$

where EF_k are the emission factors for the *k* gas monitored species (g/kWh); *ER* is the average of the instantaneous measurements for the *k* monitored gas (ppm or %v/v; measured values are divided by 10^6 or 10^2 respectively); *P* is the average pressure in standard conditions; Exh_{flow} is the volumetric flow rate of the exhaust (m^3/h); *MM* is the molar mass of the *k* monitored parameter (g/mol); *R* is the ideal gas constant (J/molK); *T* is the average temperature in standard conditions (K); *Engine_output* is the average generated engine power

(kW); $EF_{PM2.5}$ are the $PM_{2.5}$ emission factors (g/kWh); $PM_{2.5}$ is the average of the relevant instantaneous measurements (g/m^3); *DR* is the dilution ratio (25).

The scrubber requires ~80–100 kW of power, which leads to increased fuel consumption during its operation. As shown in Equations 1 and 2, the calculation of the TtW emission factors accounts for respective fuel consumption in each case (including the additional fuel consumption to operate the scrubber in case of HFO), as well as the generated engine output from both the main engine (driving the propeller) and the auxiliary engines (supporting scrubber operation in case of HFO). This approach ensures that the energy required to operate the scrubber is fully incorporated into the analysis. To enable comparison with WtT values, the HFO TtW emission factors, originally expressed in g/kWh of engine output, were converted to g/MJin using the average engine efficiency (data provided by Oldendorff). This ensures consistency in the functional unit across the analysis, allowing for direct integration and comparison of WtT and TtW impacts.

The fuel consumption in Equation 1 was measured using onboard mass flow meters, installed to monitor and ensure proper engine and vessel operation. Specifically, two mass flow meters are installed at the inlet and outlet of the circulation line of the main engine, and one mass flow meter is used for the diesel generators. These flow meters are of high accuracy, with an uncertainty of less than 0.5% of the reading. The engine output in Equation 2 for each mode was monitored and reported real-time by the relevant engine monitoring systems of the main and the auxiliary engines aboard the vessel.

The DustTrak™ DRX 8533EP instrument was factory-calibrated to ISO 12103-1, A1, Arizona test dust. Due to the significant challenges of performing gravimetric measurements

to validate DustTrak readings onboard, we conducted the $PM_{2.5}$ mass measurements using the factory calibration. After converting the measured $PM_{2.5}$ concentrations to normal conditions and calculating the $PM_{2.5}$ emission factors, we compared the results to theoretical emission factors based on the sulfur content of the fuels as reported in the IMO 4th GHG Study[26]. We observed that multiplying the measured weighted average emission factors during ME operation for MGO, VLSFO, and HFO (pre-scrubber) by a factor of 15 aligned them closely with the theoretical values: ~0.2 g/kWh for MGO, ~0.3 g/kWh for VLSFO, and ~1.4 g/kWh for HFO before the scrubber. We therefore applied this as a correction factor to the all the $PM_{2.5}$ measured emission factors. While this approach assumes that the calibration dust is the primary cause of the discrepancy, we acknowledge that additional factors, such as particle composition or size distribution, may also contribute to the observed differences. This correction allowed us to generate emission factors that are more representative of the studied fuels under real-world conditions.

NO_x emissions from internal combustion engines are defined as the sum of nitrogen monoxide (NO) and nitrogen dioxide (NO_2) emissions[14,26]. However, the employed experimental setup in this work only measured NO emissions. Based on the NO measurements, the NO_x emissions were calculated assuming a conservative 15% contribution of NO_2 to the total NO_x, which is considered as a typical NO_2 ratio for turbocharged diesel engines without aftertreatment[26]. NO and NO_2 ratios in the total NO_x emissions were expressed in the calculations through the relevant molar masses in Equation (2).

The SO_2 emissions from each fuel were also calculated based on their sulfur levels as per ISO 8178 and the IMO 4th GHG study[27]. Measurements were taken in duplicates and average values were used.

Gaseous and PM2.5 TtW emission factors (EFs) are presented per kWh, while washwater EFs are presented as concentrations to facilitate comparison with existing literature and guidelines.

Note 12 │ WtT Results

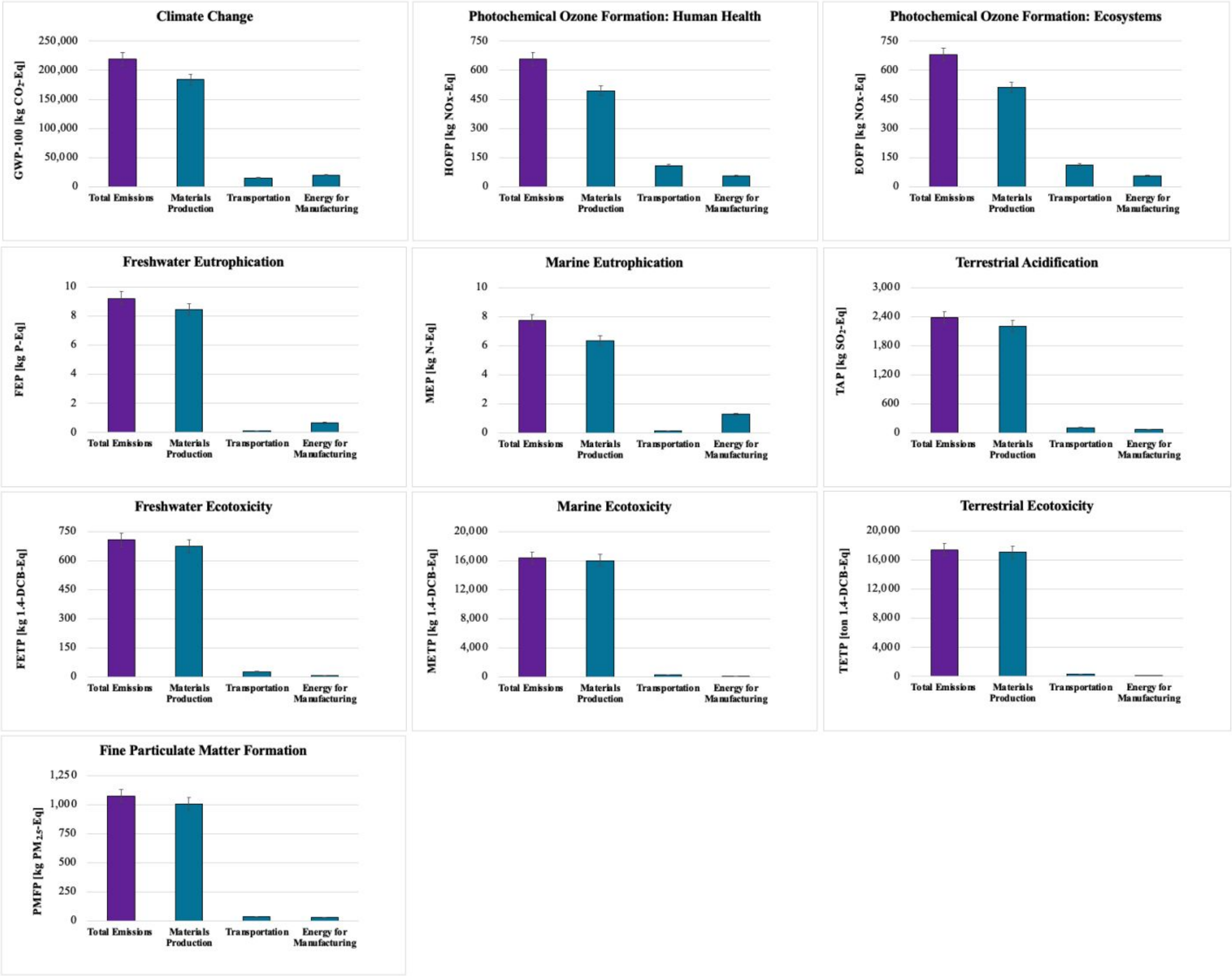

Figure S15. Scrubber WtT Impacts. GWP-100: Global Warming Potential over a 100-year time horizon; HOFP: Human health Ozone Formation Potential; EOFP: Ecosystem Ozone Formation Potential; FEP: Freshwater Eutrophication Potential; MEP: Marine Eutrophication Potential; TAP: Terrestrial Acidification Potential; FETP: Freshwater Ecotoxicity Potential; METP: Marine Ecotoxicity Potential; TETP: Terrestrial Ecotoxicity Potential; PMFP: Particulate Matter Formation Potential.

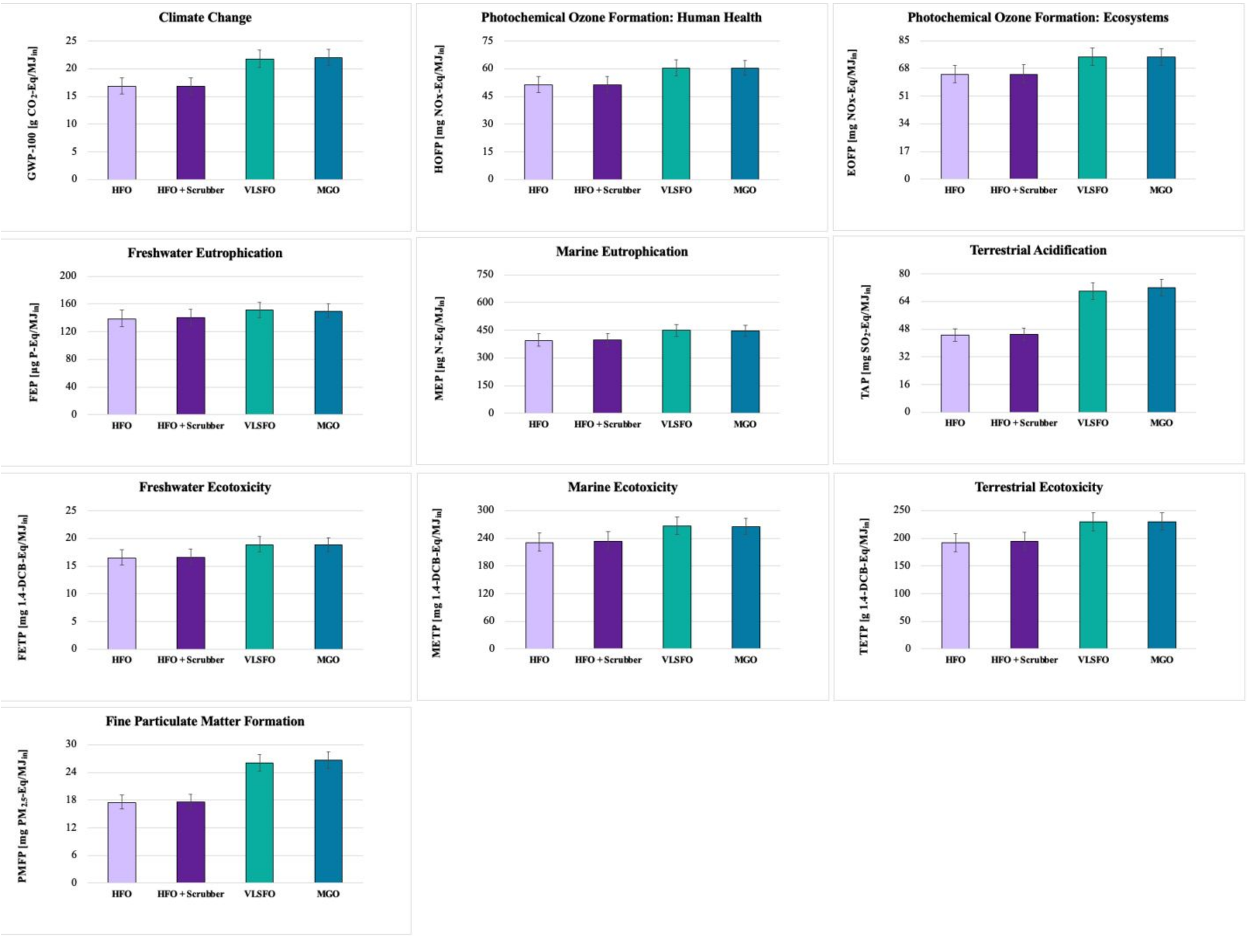

Figure S16. WtT impacts of HFO, VLSFO, MGO, and HFO with a scrubber per MJ of engine input energy. GWP-100: Global Warming Potential over a 100-year time horizon; HOFP: Human health Ozone Formation Potential; EOFP: Ecosystem Ozone Formation Potential; FEP: Freshwater Eutrophication Potential; MEP: Marine Eutrophication Potential; TAP: Terrestrial Acidification Potential; FETP: Freshwater Ecotoxicity Potential; METP: Marine Ecotoxicity Potential; TETP: Terrestrial Ecotoxicity Potential; PMFP: Particulate Matter Formation Potential.

Note 13 | TtW Results

	Idle Mode	ME @ 25%	ME @ 50%	ME @ 80%
Consumed fuel (kg/hr)	270.00	810.00	1,460.00	2,200.00
N IN GAS EMISSIONS				
NOx upstream scrubber (g/kWh)	10.16	14.49	15.96	11.40
NOx upstream scrubber (kg/h)	11.88	65.79	133.63	148.96
**N upstream the scrubber (kg/h)**	**5.41**	**29.95**	**60.83**	**67.80** (NO is assumed to be 85% and NO2 15% of total NOx)
NOx downstream scrubber (g/kWh)	8.00	11.93	12.90	9.22
NOx downstream scrubber (kg/h)	9.35	54.16	108.04	120.52
**N downstream scrubber (kg/h)**	**4.26**	**24.65**	**49.18**	**54.86**
Removed NOx (g/kWh)	2.16	2.56	3.06	2.18
**Removed N by the scrubber (kg/h)**	**1.15**	**5.29**	**11.65**	**12.94**
Reduction (%)	-0.21	-0.18	-0.19	-0.19
**Engine output (kW)**	1,169.14	4,539.11	8,372.83	13,067.16
N IN WASHWATER EMISSIONS				
Total N inlet (mg/L)	0.23	0.26	0.30	0.24
**Total N inlet (kg/h)**	**0.07**	**0.08**	**0.17**	**0.15**
Total N outlet (mg/L)	0.59	0.32	0.47	0.32
**Total N outlet (kg/h)**	**0.17**	**0.10**	**0.26**	**0.19**
Net N coming out (mg/L)	0.36	0.06	0.17	0.08
Net N coming out (mg/h)	104,400.00	19,260.00	94,350.00	48,400.00
**Net N coming out of the scrubber (kg/h)**	**0.10**	**0.02**	**0.09**	**0.05**
**ww discharge (m3/h)**	290.00	321.00	555.00	605.00
**ww discharge (L/h)**	290,000.00	321,000.00	555,000.00	605,000.00
**Fraction of Removed gas emissions**	**0.0908**	**0.0036**	**0.0081**	**0.0037**
**TOTAL N IN (kg/h)**	**5.47**	**30.03**	**60.99**	**67.95**
**TOTAL N OUT (kg/h)**	**4.43**	**24.75**	**49.44**	**55.05**

Figure S17. N mass balance calculations comparing gaseous N removed and net N released in scrubber washwater.

	Idle Mode	ME @ 25%	ME @ 50%	ME @ 80%
Consumed fuel (kg/hr)	270.00	810.00	1,460.00	2,200.00
HFO S content (%)	0.03	0.03	0.03	0.03
**Total S IN (kg/h)**	**8.32**	**24.95**	**44.97**	**67.76**
**S IN GAS & PM EMISSIONS**				
SO2 upstream of the scrubber (g/kWh)	13.89	10.73	10.49	10.13
SO2 upstream of the scrubber (kg/h)	16.24	48.72	87.81	132.32
**S upstream of the scrubber as SO2 (kg/h)**	**8.13**	**24.39**	**43.95**	**66.23**
SO2 downstream of the scrubber (g/kWh)	0.42	0.32	0.31	0.30
SO2 downstream of the scrubber (kg/h)	0.49	1.46	2.63	3.97
**S downstream of the scrubber as SO2 (kg/h)**	**0.24**	**0.73**	**1.32**	**1.99**
Removed SO2 by the scrubber (g/kWh)	13.47	10.41	10.17	9.82
**Removed S by the scrubber (kg/h)**	**7.88**	**23.65**	**42.64**	**64.25**
**Engine output (kW)**	1,169.14	4,539.11	8,372.83	13,067.16
**S IN WASHWATER EMISSIONS**				
Sulfates inlet (mg/L)	2,570.00	2,626.00	2,610.00	2,602.00
Sulphates outlet (mg/L)	2,670.00	2,850.00	2,860.00	2,900.00
Net sulfates coming out (mg/L)	100.00	224.00	250.00	298.00
Net sulfates coming out (kg/h)	29.00	71.90	138.75	180.29
**Net S coming out in sulfates (kg/h)**	**9.68**	**24.00**	**46.32**	**60.18**
Sulfites inlet (mg/L)	0.05	0.07	0.08	0.06
Sulfites outlet (mg/L)	0.06	0.08	0.08	0.07
Net sulfates coming out (mg/L)	0.01	0.01	0.00	0.01
**Net sulfites coming out (kg/h)**	**0.00**	**0.00**	**0.00**	**0.01**
outcoming flow (L/h)	290,000.00	321,000.00	555,000.00	605,000.00
**TOTAL S IN (kg/h)**	**8.32**	**24.95**	**44.97**	**67.76**
**TOTAL S OUT (kg/h)**	**9.93**	**24.74**	**47.64**	**62.18**

Figure S18. S mass balance calculations comparing gaseous S removed and net S released in scrubber washwater.

	Idle Mode	ME @ 25%	ME @ 50%	ME @ 80%
Consumed fuel (kg/hr)	270.00	810.00	1,460.00	2,200.00
HFO C content (%)	0.85	0.85	0.85	0.85
**Total C IN CONSUMED FUEL (kg/h)**	**230.53**	**691.58**	**1,246.55**	**1,878.36**
**C IN GAS EMISSIONS**				
CO2 upstream scrubber (g/kWh)	729.88	564.38	550.44	531.18
CO2 upstream scrubber (kg/h)	853.34	2,561.78	4,608.75	6,940.98
**C upstream the scrubber as CO2 (kg/h)**	**232.87**	**699.09**	**1,257.69**	**1,894.14**
CO2 downstream scrubber (g/kWh)	730.05	563.91	550.04	530.84
CO2 downstream scrubber (kg/h)	853.53	2,559.64	4,605.38	6,936.64
**C downstream scrubber as CO2 (kg/h)**	**232.92**	**698.51**	**1,256.77**	**1,892.96**
CO upstream scrubber (g/kWh)	2.49	0.72	0.40	0.36
CO upstream scrubber (kg/h)	2.91	3.28	3.38	4.72
**C upstream the scrubber as CO (kg/h)**	**1.25**	**1.41**	**1.45**	**2.02**
CO downstream scrubber (g/kWh)	1.04	0.60	0.35	0.31
CO downstream scrubber (kg/h)	1.22	2.73	2.89	4.08
**C downstream scrubber as CO (kg/h)**	**0.52**	**1.17**	**1.24**	**1.75**
**Engine output (kW)**	1,169.14	4,539.11	8,372.83	13,067.16
**TOTAL C IN (kg/h)**	**234.12**	**700.50**	**1,259.14**	**1,896.16**
**TOTAL C OUT (kg/h)**	**233.44**	**699.68**	**1,258.01**	**1,894.71**

Figure S19. C mass balance calculations comparing gaseous C upstream and downstream of the scrubber.

Over time, a small amount of solid residue accumulates on the scrubber walls and is periodically collected and properly disposed of onshore (Figure S20). Approximately 15 kg of residue is generated over 30 days of scrubber operation. Over the course of a year (200 days of operation), around 100 kg of solid residue is being generated. This amount is negligible when compared to the vessel's total incoming energy (~316 million MJ annually), equating to approximately 0.0003 g/MJ_{in}. As such, it was not included in the LCA.

Results of the elemental analysis of the solid residue are presented in (Figures S21 and S22).

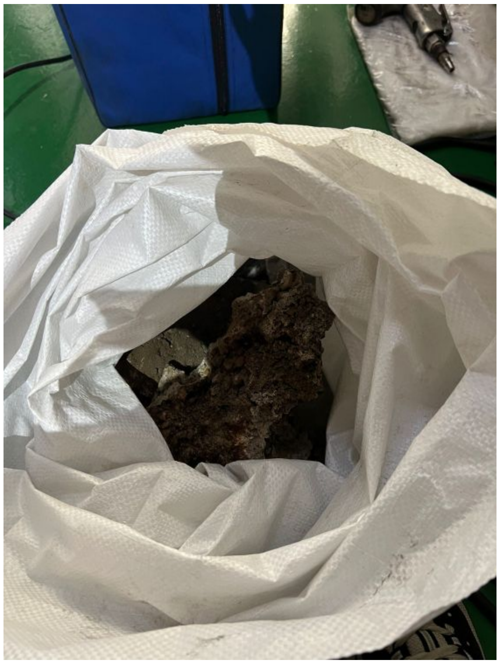

Figure S20. The scrubber solid residue.

Analytical Report

Control Union Vessel Performance Centre BV
Attn: . .
Boompjes 270
3011 XZ Rotterdam
Netherlands

Reportnr.	: **1815325 version 1**		
Sample Arrival Date	: 12-Dec-2023 18:22	Sampling Date	* : 12-Dec-2023
ReportDate Version	: **22-Jan-2024 16:01**	Samplesize (kg)	: 2
Packing	: Plastic, ambient	Seal / Seal Code	: No /

Sample information *

Product specification : Solid scrubber deposits

*** Information supplied by customer (TLR takes no responsibility for this information).**

Composition Determination

Common

**Parameter**	**Result (as received)**		
Carbon (C)	1,8	%	R
Nitrogen.	< 0,05	%	R

Metal and other elements

**Parameter**	**Result (as received)**		
Hg (Mercury)	< 0,05	mg/kg	R
V total	117	mg/kg	R
Mn total	< 1,3	mg/kg	R
Co total	4,2	mg/kg	R
Ni total	85,7	mg/kg	R
Cu total	< 0,9	mg/kg	R
Zn total	20,0	mg/kg	R
As total	0,3	mg/kg	R
Se total	< 0,5	mg/kg	R
Cd total	< 0,04	mg/kg	R
Sb total	< 2,5	mg/kg	
Ba total	< 1,3	mg/kg	R
Pb total	< 2,0	mg/kg	R
Na total	330000	mg/kg	R
Mg total	32400	mg/kg	R
Sn total	< 1,0	mg/kg	R

Figure S21. Elemental analysis of the solid residue (1/2).

Analytical Report

Reportnr.	: **1815325 version 1**		
Sample Arrival Date	: 12-Dec-2023 18:22	Sampling Date	* : 12-Dec-2023
ReportDate Version	: **22-Jan-2024 16:01**	Samplesize (kg)	: 2
Packing	: Plastic, ambient	Seal / Seal Code	: No /

ANNEX

Method Descriptions

Composition Determination

Metal and other elements

**Method Description**	**Method Code**
Determination of Mercury (Hg); Hg-analyser<br>Animalfeed/feedingstuff : eq. NEN-EN16277 Food : eq. NEN-EN15763	.
Determination of metals with inductively coupled plasma mass spectrometry (ICP-MS)	own method

Abbreviations:
acc: in accordance with
eq: Equivalent to

Figure S22. Elemental analysis of the solid residue (2/2).

Out of the 23 metals tested (Table S6) only the following 10 showed statistically significant increases in the scrubber washwater: V, Fe, Ni (Figure 6C), and Al, Cr, Co, Cu, Pb, Hg, Zn (Figure S23).

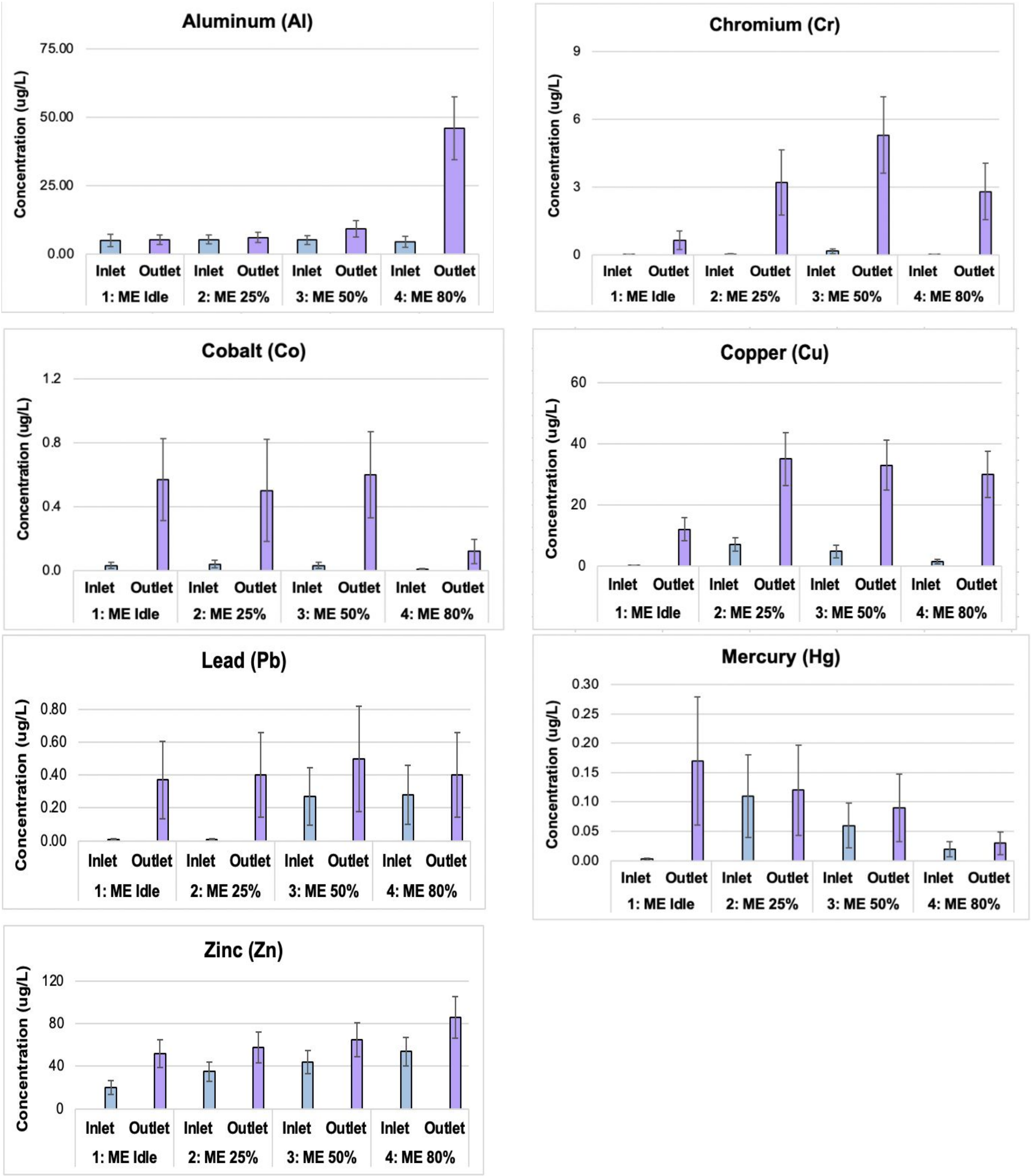

Figure S23. Metals in incoming seawater (inlet) and scrubber washwater (outlet).

The maximum observed values for the 10 metals were compared with the US EPA 40 CFR, Part 437 - Centralized Waste Treatment Point Source Category limits[28], which concern discharges from centralized facilities treating a wide range of industrial effluents (Table S7). In addition, they were compared with the maximum emission levels for direct discharge to receiving water bodies from the BAT Reference Document for Waste Treatment under the EU Industrial Emissions Directive[29]. Iron concentrations, not covered by the above-mentioned standards, were compared to the US EPA maximum daily limits concerning discharges from steam electric power generating operations[30].

Moreover, assuming a conservative 1,000-fold dilution of the maximum observed concentrations, we compared the expected final concentrations of scrubber pollutants in receiving water bodies with the EU environmental quality standards (EQS) for priority pollutants[31-34] and the US EPA water quality criteria for aquatic life in seawater[35,36] (Table S7). We focused on the chronic limits for saltwater, as they are more stringent and address long-term, cumulative exposure effects.

Table S7. Maximum observed and diluted washwater metal concentrations compared to US and EU industrial wastewater discharge regulations and environmental criteria.

Metal	Max metal conc. in scrubber washwater discharge (μg/L)	Max limits for industrial wastewater discharge (μg/L)	Metal conc. after 1,000-fold dilution (μg/L)	US EPA Aquatic Life Criteria (μg/L; chronic limits)[35]	EU Environmental Quality Standards (EQS) (μg/L)[31,32]
Aluminum (Al)	46 ± 12	-	0.046	0.63[*36]	-
Chromium (Cr)	5.3 ± 1.7	746[28]; 150[29]	0.0053	50[**]	-
Cobalt (Co)	0.6 ± 0.3	192[28]	0.0006	-	-
Copper (Cu)	35 ± 9	500[28,29]	0.035	3.1	-
Iron (Fe)	60 ± 15	1,000[30]	0.06	-	-
Lead (Pb)	0.4 ± 0.3	350[28]; 100[29]	0.0004	8.1	Annual avg: 1.3; Max limit: 14
Mercury (Hg)	0.2 ± 0.1	2.34[28]; 5[29]	0.0002	0.94	Max limit: 0.07 (0.01[33])
Nickel (Ni)	21 ± 6.7	3,950[28]; 1,000[29]	0.021	8.2	Annual average: 8.6 (3.1[34]); Max limit: 34 (8.2[34])
Vanadium (V)	81 ± 18	218[28]	0.081	-	-
Zinc (Zn)	52 ± 13	497[28]; 2,000[29]	0.052	81	-

[*]Chronic limit for freshwater; US EPA has not established specific aluminum criteria for saltwater[35].
[**]Chromium (VI).

To put the measured values for organic compounds in scrubber washwater into perspective, we compared them with relevant US and EU limits for industrial wastewater discharge (Table S8). Specifically, the maximum observed values for PAHs and BTEX were compared with the maximum daily limits for wastewater discharges from the manufacturing of organic chemicals, plastics, and synthetic fibers in the US[37]. We considered BAT effluent limits for direct discharge point sources that do not use end-of-pipe biological treatment to enable a comparison with some of the strictest US industrial limits for these pollutants. For oil and grease, we considered the strictest maximum daily limit for discharges from petroleum refining operations in the US[38].

Additionally, we considered BAT-associated emission limits for the hydrocarbon oil index for direct industrial discharges to receiving water bodies in the EU[29]. Moreover, assuming a conservative 1,000-fold dilution in open sea, we compared the expected final concentrations of organic pollutants in the receiving water bodies with the EU EQS for priority pollutants[31,32] and the US EPA national recommended water quality criteria for aquatic life[35] (Table S8).

Table S8. Maximum observed and diluted concentrations of organic pollutants compared to US and EU industrial wastewater discharge regulations and environmental criteria.

Organic Pollutant	Max conc. in scrubber washwater discharge (µg/L)	Max limits for industrial wastewater discharge (µg/L)	Conc. after 1,000-fold dilution (µg/L)	US EPA Aquatic Life Criteria (µg/L)[35]	EU EQS (µg/L)[31,32]
Fluoranthene	0.01 ± 0.008	54[37]	0.00001	-	0.0063* (0.000762[40]**); 0.12***
Fluorene	0.05 ± 0.03	47[37]	0.00005	-	-
Phenanthrene	0.25 ± 0.11	47[37]	0.00025	-	-
Total PAHs	0.31 ± 0.12	-	0.00031	-	-
Toluene	7.60 ± 3.42	74[37]	0.00760	-	-
Oil & grease	<1,000	15,000[37]	<1	1[38]	-
Hydrocarbon oil index (C10-C40)	50 ± 15	500-10,000[29]	0.05	-	0.4*; 1.4*** for C10-13 chloroalkanes

*Annual average EQS value.
**Proposed annual average EQS value.
***Maximum allowable EQS concentration.

Note 14 | Comparison of Scrubber Washwater with Prior Studies

Total N, P, metal and hydrocarbon concentrations at the inlet were compared to native levels reported in the literature from field studies in China's coastal waters, and particularly in the East and South China Sea areas, where our study took place[41,42]. Apart from hydrocarbons which were lower than reported values, the measured inlet values were well within the reported ranges of native concentrations, tending towards the lower end. This suggests that the vessel's pumping equipment did not introduce contamination to the incoming seawater.

Measured metal and organic compound concentrations at the outlet were within the ranges reported for open-loop scrubbers aboard ocean-going vessels[43-48]. Maximum Ni and V values observed in this study are lower than those reported in[43-45,47,48], whereas maximum Cu values are higher compared to[44-46]. Maximum PAH concentrations reported in this study align well with the average total outlet concentrations reported in[45], which sampled 50 vessels, but are orders of magnitude lower than those reported by[43-44,48], primarily due to naphthalene and phenanthrene. In addition, DHI, (2021)[46] and Magnusson et al., (2018)[44] reported significantly higher BTEX and total hydrocarbon concentrations, respectively, compared to our study. It should be noted that, inlet PAH concentrations in Jalkanen et al., (2024)[43] were significantly high, indicating potential seawater contamination, while in Magnusson et al., (2018)[44], no incoming seawater concentrations are reported. These limitations, along with the lack of detailed information on the relevant engines and operating conditions under which sampling was conducted in most studies, make it difficult to equitably compare our values with those reported.

Note 15 | WtW Results

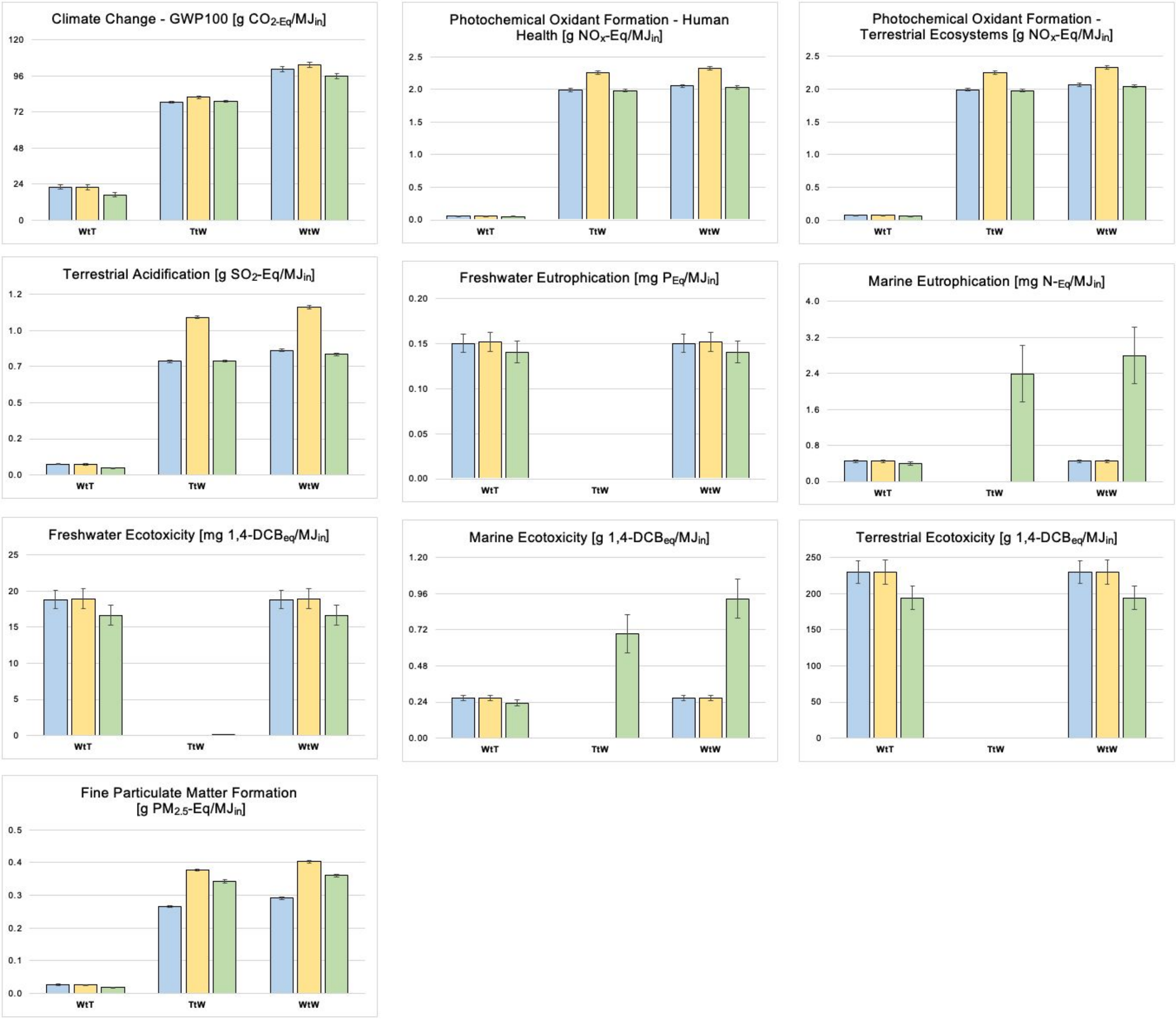

Figure S24. WtW impacts of HFO, VLSFO, MGO, and HFO with a scrubber per MJ of engine input energy. GWP-100: Global Warming Potential over a 100-year time horizon; FEP: Blue bars: MGO; Yellow bars: VLSFO; Green bars: HFO + Scrubber (post-scrubber values).

Limited LCA studies exist that compare the WtW impacts of the three considered fuels, focusing mostly on climate change and acidification impacts, and often not being informed by on-board measurements under similar conditions. In impact categories related to atmospheric releases, TtW impacts are typically dominant. The reported GHG WtW

emissions of HFO with a scrubber are comparable to those of MGO, with differences fluctuating within a range of ± 5%, being consistent with our findings[4,27,49,50]. Brynolf et al., (2014)[51] reported higher fine PM formation impacts for HFO with a scrubber compared to MGO as well as similar photochemical ozone formation, acidification, and eutrophication WtW impacts between the two fuels, aligning with our results. However, due to the lack of uncertainty ranges and significant differences in underlying methodologies, direct comparisons with our values are not feasible.

Ytreberg et al., (2021)[52] employed the ReCiPe methodology[12] to assess the marine ecotoxicity impacts of various shipping discharges, including scrubber washwater. They report a marine ecotoxicity potential for open-loop scrubbers of 0.23 ± 0.11 kg 1.4-Dichlorobenzene-Eq/m^3, which is an order of magnitude higher than our value per m^3, *i.e.,* 0.07 ± 0.01 kg 1.4-Dichlorobenzene-Eq/m^3. Despite using the same characterization factors, they considered the total concentrations of pollutants in scrubber washwater, whereas our approach subtracts the concentrations of incoming seawater to avoid double counting pollutants in the receiving water bodies. Additionally, the pollutant concentrations in their study were higher. This methodological difference highlights the need for a standardized LCA approach to more accurately assess the impacts of scrubber discharges.

REFERENCES

(1) Wernet, G.; Bauer, C.; Steubing, B.; Reinhard, J.; Moreno-Ruiz, E.; Weidema, B. The Ecoinvent Database Version 3 (Part I): Overview and Methodology. *Int. J. Life Cycle Assess.* **2016**, *21* (9), 1218–1230. https://doi.org/10.1007/s11367-016-1087-8.

(2) *Marine Gasoil (MGO)*. Oiltanking. https://www.oiltanking.com/en/news-info/glossary/marine-gasoil-mgo.html (accessed 2024-09-15).

(3) Vermeire, M. *Everything You Need to Know about Marine Fuels*; IDU2011737 8/21; Chevron, Chevron Marine Products: Ghent, Belgium, 2021; p 32.

(4) Pavlenko, N.; Comer, B.; Zhou, Y.; Clark, N.; Rutherford, D. *The Climate Implications of Using LNG as a Marine Fuel*; ICCT WORKING PAPER 2020-02; The International Council on Clean Transportation (ICCT), 2020.

(5) *IMO2020 fuel oil sulphur limit - cleaner air, healthier planet*. https://www.imo.org/en/MediaCentre/PressBriefings/pages/02-IMO-2020.aspx (accessed 2024-09-08).

(6) Marsh, P.; Shamray, A. *Buying Fuel on Calorific Value as Means to Achieve Savings*; Integr8 Fuels, Research and Advisory Services Division, 2019; p 4.

(7) *Fuels - Higher and Lower Calorific Values*. https://www.engineeringtoolbox.com/fuels-higher-calorific-values-d_169.html (accessed 2024-09-09).

(8) Stripple, H.; Zhang, Y. *Scrubbers: Closing the Loop Activity 3: Task 4 Evaluation of Exhaust Gas Scrubber Systems for Ship Applications from a System Perspective*; B 2321; IVL Swedish Environmental Research Institute: Stockholm, Sweden, 2019; p 80.

(9) Andersson, K.; Jeong, B.; Jang, H. Life Cycle and Cost Assessment of a Marine Scrubber Installation. *J. Int. Marit. Saf. Environ. Aff. Shipp.* **2020**, *4* (4), 162–176. https://doi.org/10.1080/25725084.2020.1861823.

(10) Faber, J.; Kleijn, A.; Jaspers, D. *Comparison of CO2 Emissions of MARPOL Annex VI Compliance Options in 2020*; 20.190191E.091; Delft, CE Delft, 2020.

(11) Stathatou, P. M.; Bergeron, S.; Fee, C.; Jeffrey, P.; Triantafyllou, M.; Gershenfeld, N. Towards Decarbonization of Shipping: Direct Emissions & Life Cycle Impacts from a Biofuel Trial Aboard an Ocean-Going Dry Bulk Vessel. *Sustain. Energy Fuels* **2022**, *6* (7), 1687–1697. https://doi.org/10.1039/D1SE01495A.

(12) Huijbregts, M. A. J.; Steinmann, Z. J. N.; Elshout, P. M. F.; Stam, G.; Verones, F.; Vieira, M. D. M.; Hollander, A.; Zijp, M.; van Zelm, R. *ReCiPe 2016 v1.1 A Harmonized Life Cycle Impact Assessment Method at Midpoint and Endpoint Level Report I: Characterization*; RIVM Report 2016-0104a; National Institute for Public Health and the Environment: The Netherlands, 2016; p 201.

(13) Aakko-Saksa, P. T.; Lehtoranta, K.; Kuittinen, N.; Järvinen, A.; Jalkanen, J.-P.; Johnson, K.; Jung, H.; Ntziachristos, L.; Gagné, S.; Takahashi, C.; Karjalainen, P.; Rönkkö, T.; Timonen, H. Reduction in Greenhouse Gas and Other Emissions from Ship Engines: Current Trends and Future Options. *Prog. Energy Combust. Sci.* **2023**, *94*, 101055. https://doi.org/10.1016/j.pecs.2022.101055.

(14) The International Council and on Combustion Engines (CIMAC). *Guide to Diesel Exhaust Emissions Control of NOx, SOx, Particulates, Smoke and CO2: Seagoing Ships and Large Stationary Diesel Power Plants*; CIMAC Working Group “Exhaust Emissions Control”: Frankfurt, Germany, 2008; p 36.

https://www.cimac.com/cms/upload/Publication_Press/Recommendations/Recommendation_28.pdf.
(15) *P2000 In-Situ CEM | Analyser Product Range | Protea Ltd*. https://www.protea.ltd.uk/p2000-in-situ-cem (accessed 2024-09-09).
(16) International Organization for Standardization (ISO). INTERNATIONAL STANDARD ISO 8178-4: Reciprocating Internal Combustion Engines: Exhaust Emission Measurement. Part 4: Steady-State and Transient Test Cycles for Different Engine Applications, 2020.
(17) *Scrubber Water*. Naias Labs S.A. https://www.naiaslabs.com/scrubber-water/ (accessed 2024-09-09).
(18) *ISOLAB English Catalog 2023-2025*. https://isolab.de/englishcatalog/#150 (accessed 2024-09-11).
(19) *2100Q IS Portable Turbidimeter (LED), 0-1000 FNU*. https://www.hach.com/p-2100q-portable-turbidimeters/2100QIS01 (accessed 2024-09-11).
(20) *Colorimetric test kit VISOCOLOR HE Chlorine, for low concentrations MN | MACHEREY-NAGEL*. https://www.mn-net.com/us/colorimetric-test-kit-visocolor-he-chlorine-for-low-concentrations-920015?c=5151 (accessed 2024-09-11).
(21) *Naias Labs | Quality control Quantity Inspections for bunker, fuel & lubricant*. Naias Labs S.A. https://www.naiaslabs.com/ (accessed 2024-09-18).
(22) Chu-Van, T.; Ristovski, Z.; Pourkhesalian, A. M.; Rainey, T.; Garaniya, V.; Abbassi, R.; Kimball, R.; Luong Cong, N.; Jahangiri, S.; Brown, R. J. A Comparison of Particulate Matter and Gaseous Emission Factors from Two Large Cargo Vessels during Manoeuvring Conditions. *Energy Rep.* **2019**, *5*, 1390–1398. https://doi.org/10.1016/j.egyr.2019.10.001.
(23) Chu-Van, T.; Ristovski, Z.; Pourkhesalian, A. M.; Rainey, T.; Garaniya, V.; Abbassi, R.; Jahangiri, S.; Enshaei, H.; Kam, U.-S.; Kimball, R.; Yang, L.; Zare, A.; Bartlett, H.; Brown, R. J. On-Board Measurements of Particle and Gaseous Emissions from a Large Cargo Vessel at Different Operating Conditions. *Environ. Pollut.* **2018**, *237*, 832–841. https://doi.org/10.1016/j.envpol.2017.11.008.
(24) International Association of Maritime Universities (IAMU). *Development of a Methodology to Measure and Assess Ship Emissions*; Tokyo, Japan, 2016; p 70.
(25) Khan, M. Y.; Russell, R. L.; Welch, W. A.; Cocker, D. R. I.; Ghosh, S. Impact of Algae Biofuel on In-Use Gaseous and Particulate Emissions from a Marine Vessel. *Energy Fuels* **2012**, *26* (10), 6137–6143. https://doi.org/10.1021/ef300935z.
(26) *Gaseous Emissions*. https://dieselnet.com/tech/emissions_gas.php (accessed 2024-09-18).
(27) Faber, J.; Hanayama, S.; Zhang, S.; Pereda, P.; Comer, B.; Hauerhof, E.; Schim van der Loeff, W.; Smith, T.; Zhang, Y.; Kosaka, H.; Adachi, M.; Bonello, J.-M.; Galbraith, C.; Gong, Z.; Hirata, K.; Hummels, D.; Kleijn, A.; Lee, D. S.; Liu, Y.; Lucchesi, A.; Mao, X.; Muraoka, E.; Osipova, L.; Qian, H.; Rutherford, D.; Suárez de la Fuente, S.; Yuan, H.; Velandia Perico, C.; Wu, L.; Sun, D.; Yoo, D.-H.; Xing, H. *Fourth IMO GHG Study 2020*; International Maritime Organization (IMO): London, 2021; p 524.
(28) United States Environmental Protection Agency (EPA). *40 CFR Part 437-The Centralized Waste Treatment Point Source Category*; 2000; Vol. 40. https://www.ecfr.gov/current/title-40/part-437 (accessed 2024-09-17).
(29) Pinasseau, A.; Zerger, B.; Roth, J.; Canova, M.; Roudier, S. *Best Available Techniques (BAT) Reference Document for Waste Treatment: Industrial Emissions Directive 2010/75/EU (Integrated Pollution Prevention and Control).*; Policy Report EUR 29362 EN;

Publications Office of the European Union: Luxembourg, 2018; p 851. https://data.europa.eu/doi/10.2760/407967 (accessed 2024-09-05).
(30) United States Environmental Protection Agency (EPA). *40 CFR Part 423-Steam Electric Power Generating Point Source Category*; Vol. 40. https://www.ecfr.gov/current/title-40/part-423 (accessed 2024-09-17).
(31) European Commission. *Directive 2000/60/EC of the European Parliament and of the Council of 23 October 2000 Establishing a Framework for Community Action in the Field of Water Policy*; 2000; Vol. 327, pp 1–73. https://eur-lex.europa.eu/eli/dir/2000/60/oj (accessed 2024-09-17).
(32) European Commission. *Directive 2008/105/EC of the European Parliament and of the Council of 16 December 2008 on Environmental Quality Standards in the Field of Water Policy, Amending and Subsequently Repealing Council Directives 82/176/EEC, 83/513/EEC, 84/156/EEC, 84/491/EEC, 86/280/EEC and Amending Directive 2000/60/EC of the European Parliament and of the Council*; 2008; Vol. 348, pp 84–97. http://data.europa.eu/eli/dir/2008/105/oj/eng (accessed 2024-09-17).
(33) European Commission. Common Implementation Strategy for the Water Framework Directive, Environmental Quality Standards (EQS) Substance Data Sheet: Priority Substance No. 21, Mercury and Its Compounds-CAS-No. 7439-97-6, 2023. https://circabc.europa.eu/ui/group/9ab5926d-bed4-4322-9aa7-9964bbe8312d/library/7ac7374a-d259-47c0-8ab7-af3043769a9f?p=1&n=10&sort=modified_DESC (accessed 2024-09-17).
(34) European Commission. Nickel_Final EQS Dossier, 2023. https://circabc.europa.eu/ui/group/9ab5926d-bed4-4322-9aa7-9964bbe8312d/library/208fe9a5-985b-43d6-b2ae-5f7c14c5581a?p=1&n=10&sort=modified_DESC (accessed 2024-09-17).
(35) US EPA. *National Recommended Water Quality Criteria - Aquatic Life Criteria Table*. https://www.epa.gov/wqc/national-recommended-water-quality-criteria-aquatic-life-criteria-table (accessed 2024-09-17).
(36) United States Environmental Protection Agency (EPA). *Fact Sheet: Final 2018 Aquatic Life Ambient Water Quality Criteria for Aluminum in Freshwaters*; EPA 822-F-18-003; US EPA, Office of Water, 2018.
(37) United States Environmental Protection Agency (EPA). *40 CFR Part 414-Organic Chemicals, Plastics, and Synthetic Fibers*; Vol. 40. https://www.ecfr.gov/current/title-40/part-414 (accessed 2024-09-18).
(38) United States Environmental Protection Agency (EPA). *40 CFR Part 419-Petroleum Refining Point Source Category*; Vol. 40. https://www.ecfr.gov/current/title-40/part-419 (accessed 2024-09-18).
(39) United States Environmental Protection Agency (EPA). *Quality Criteria for Water*; EPA 440/5-86-001; US EPA, Office of Water: Washington DC, 1986; p 395. https://www.epa.gov/sites/default/files/2018-10/documents/quality-criteria-water-1986.pdf (accessed 2024-09-18).
(40) European Commission. Fluoranthene-EQS Dossier, 2023. https://circabc.europa.eu/ui/group/9ab5926d-bed4-4322-9aa7-9964bbe8312d/library/2733a034-903c-45eb-9df4-7efe70b06053?p=1&n=10&sort=modified_DESC (accessed 2024-09-18).

(41) Wu, J.; Lu, J.; Zhang, C.; Zhang, Y.; Lin, Y.; Xu, J. Pollution, Sources, and Risks of Heavy Metals in Coastal Waters of China. *Hum. Ecol. Risk Assess. Int. J.* **2020**, *26* (8), 2011–2026. https://doi.org/10.1080/10807039.2019.1634466.
(42) Gong, S.; Liu, W.; Li, Y.; Zhang, J.; Chen, C.; Fu, J. Distribution Characteristics and Source Tracing of Petroleum Hydrocarbons in the Northeastern South China Sea. *Chin. Chem. Lett.* **2020**, *31* (10), 2854–2858. https://doi.org/10.1016/j.cclet.2020.06.020.
(43) Jalkanen, J.-P.; Fridell, E.; Kukkonen, J.; Moldanova, J.; Ntziachristos, L.; Grigoriadis, A.; Moustaka, M.; Fragkou, E.; Tseges, G.; Maragkidou, A.; Sofiev, M.; Hänninen, R.; Grönholm, T.; Palamarchuk, J.; Majamäki, E.; Winiwarter, W.; Gueret, S.; Sokhi, R. S.; Kumar, S.; Alyuz Ozdemir, U.; Kolovoyiannis, V.; Zervakis, V.; Mazioti, A.-A.; Krasakopoulou, E.; Hassellöv, I.-M.; Lunde Hermansson, A.; Ytreberg, E.; Williams, I.; Hudson, M.; Zapata Restrepo, L.; Hole, L. R.; Aghito, M.; Breivik, O.; Petrovic, M.; Gross, M.; Rodriguez-Mozaz, S.; Neophytou, M.; Monteiro, A.; Russo, M. A.; Oikonomous, F.; Gondikas, A.; Marcomini, A.; Giubilato, E.; Calgaro, L.; Jaakkola, J. J. K.; Shiue, I.; Kiihamäki, S.-P.; Broström, G.; Hassellöv, M.; Kaitaranta, J.; Granberg, M.; Magnusson, K. *Environmental Impacts of Exhaust Gas Cleaning Systems in the Baltic Sea, North Sea, and the Mediterranean Sea Area*; Finnish Meteorological Institute, 2024. https://doi.org/10.35614/isbn.9789523361898.
(44) Magnusson, K.; Thor, P.; Granberg, M. Scrubbers: Closing the Loop.
(45) Bureau Veritas (Commodities Division). *Assessment of Analytical Results from EGCS Wash-Water Discharge Samples on Various Vessels*; 2021; p 35.
(46) DHI Water & Environment. *Ecotoxicity Testing and Risk Assessment of Wash Water from Open Loop Scrubbers*; Final report / 11826102; Exhaust Gas Cleaning Systems Association (EGCSA), 2021; p 73. https://www.egcsa.com/wp-content/uploads/EGCSCAFinalreport_11826102_2021.06.08.pdf (accessed 2024-09-18).
(47) Koski, M.; Stedmon, C.; Trapp, S. Ecological Effects of Scrubber Water Discharge on Coastal Plankton: Potential Synergistic Effects of Contaminants Reduce Survival and Feeding of the Copepod Acartia Tonsa. *Mar. Environ. Res.* **2017**, *129*, 374–385. https://doi.org/10.1016/j.marenvres.2017.06.006.
(48) Kjølholt, J.; Aakre, S.; Jürgensen, C.; Lauridsen, J. *Assessment of Possible Impacts of Scrubber Water Discharges on the Marine Environment*; Environmental Project No. 1431, 2012; Danish Ministry of the Environment, Environmental Protection Agency: Denmark, 2012; p 93. https://www2.mst.dk/udgiv/publications/2012/06/978-87-92903-30-3.pdf (accessed 2024-09-18).
(49) Kasseris, E.; Wang, D.; Zhang, Y.; Adams, E.; Heywood, J. *Environmental Impact Assessment of Alternatives to Meet the Low Sulfur Marine Fuel Mandate*; Massachusetts Institute of Technology: Cambridge, MA, 2019.
(50) Comer, B.; Osipova, L. *Accounting for Well-to-Wake Carbon Dioxide Equivalent Emissions in Maritime Transportation Climate Policies*; 2021.
(51) Brynolf, S.; Magnusson, M.; Fridell, E.; Andersson, K. Compliance Possibilities for the Future ECA Regulations through the Use of Abatement Technologies or Change of Fuels. *Transp. Res. Part Transp. Environ.* **2014**, *28*, 6–18. https://doi.org/10.1016/j.trd.2013.12.001.
(52) Ytreberg, E.; Åström, S.; Fridell, E. Valuating Environmental Impacts from Ship Emissions – The Marine Perspective. *J. Environ. Manage.* **2021**, *282*, 111958. https://doi.org/10.1016/j.jenvman.2021.111958.
